# Iterative machine learning-based chemical similarity search to identify novel chemical inhibitors

**DOI:** 10.1186/s13321-023-00760-6

**Published:** 2023-09-23

**Authors:** Prasannavenkatesh Durai, Sue Jung Lee, Jae Wook Lee, Cheol-Ho Pan, Keunwan Park

**Affiliations:** 1 Natural Product Informatics Research Center, Korea Institute of Science and Technology, Gangneung, 25451 Republic of Korea; 2 Natural Product Research Center, Korea Institute of Science and Technology, Gangneung, 25451 Republic of Korea; 3 Department of YM-KIST Bio-Health Convergence, Yonsei University, Wonju, 26493 Republic of Korea

**Keywords:** MEK inhibitor, Evolutionary chemical binding similarity, Virtual screening, Hit identification, Drug design

## Abstract

Machine learning-based chemical screening has made substantial progress in recent years. However, these predictions often have low accuracy and high uncertainty when identifying new active chemical scaffolds. Hence, a high proportion of retrieved compounds are not structurally novel. In this study, we proposed a strategy to address this issue by iteratively optimizing an evolutionary chemical binding similarity (ECBS) model using experimental validation data. Various data update and model retraining schemes were tested to efficiently incorporate new experimental data into ECBS models, resulting in a fine-tuned ECBS model with improved accuracy and coverage. To demonstrate the effectiveness of our approach, we identified the novel hit molecules for the mitogen-activated protein kinase kinase 1 (MEK1). These molecules showed sub-micromolar affinity (Kd 0.1–5.3 μM) to MEKs and were distinct from previously-known MEK1 inhibitors. We also determined the binding specificity of different MEK isoforms and proposed potential docking models. Furthermore, using de novo drug design tools, we utilized one of the new MEK inhibitors to generate additional drug-like molecules with improved binding scores. This resulted in the identification of several potential MEK1 inhibitors with better binding affinity scores. Our results demonstrated the potential of this approach for identifying novel hit molecules and optimizing their binding affinities.

**Supplementary Information:**

The online version contains supplementary material available at 10.1186/s13321-023-00760-6.

## Introduction

In modern drug discovery, machine learning-based virtual screening (VS) has become an inevitable approach for identifying biologically active compounds in vast chemical libraries. For example, novel antibiotics were discovered using a deep neural network model that was trained using a large database of chemical structures and their activity against bacterial strains [1]. A generative tensorial reinforcement learning (GENTRL) model developed by Insilico Medicine led to the discovery of novel potent DDR1 kinase inhibitors in a short time period [2]. Machine learning methods have also been applied to for different processes, including drug discovery for Alzheimer’s disease [3], target identification in cancer [4], and prediction of toxicity [5]. These studies demonstrated the potential of machine learning-based VS methods for drug discovery. In line with these developments, we recently developed a machine learning-based evolutionary chemical binding similarity (ECBS) method [6], a ligand similarity-based VS method, that leverages evolutionarily conserved target-binding properties embedded in chemical structures for more accurate hit identification. Follow-up studies have revealed that ECBS can help identify effective inhibitors of several drug targets [6–8].

Despite recent advancements, machine learning models trained on available public protein–ligand interaction data often result in a high fraction of retrieved compounds that are not novel (i.e., structurally close to known active compounds) and generate low prediction scores and high uncertainty for unseen new chemical scaffolds [9–12]. Scaffold hopping is a strategy for discovering novel active scaffolds; however, it requires sufficient information on structurally diverse compounds that specifically interact with targets [13].

To address this issue, we proposed a strategy that leverages experimental validation data from an initial screening result to optimize an ECBS-based screening model and identify a new chemical space. Because ECBS is based on classification similarity learning for chemical pairs rather than individual compounds, we evaluated different chemical pairing schemes to define optimal evolutionarily related chemical pairs (ERCPs) and assessed the value of including new experimental data for prediction refinement. We assumed that incorporating newly identified active compounds predicted by an initial ECBS model would expand the searchable space of the prediction model and that failed prediction data (false positives) would fine-tune the prediction model and improve accuracy.

To test our strategy, we selected mitogen-activated protein kinase kinase 1 (MEK1) as a target to discover novel hit molecules. Most MEK inhibitors have been developed to target the ERK pathway, which is involved in many cancers, by controlling undesirable cell growth and survival [14]. In particular, the development of MEK1 and MEK2 inhibitors has been more extensive than that of other MEKs because of their crucial roles in biological processes. In contrast, MEK5 has been linked to the development of the cardiovascular system and cancer, and a combination of MEK1/2 and MEK5 inhibitors may offer a combinatorial therapy [15]. Various resistance mechanisms involving receptor tyrosine kinases and the PI3K/AKT pathway have been discovered for most MEK inhibitors [16], emphasizing the need for new MEK inhibitors [14]. In addition to the therapeutic potential of developing new MEK inhibitors [17], competitive binding assay data on MEK1 that can be used to refine an initial ECBS model are available from our recent study [8].

**Fig. 1:**
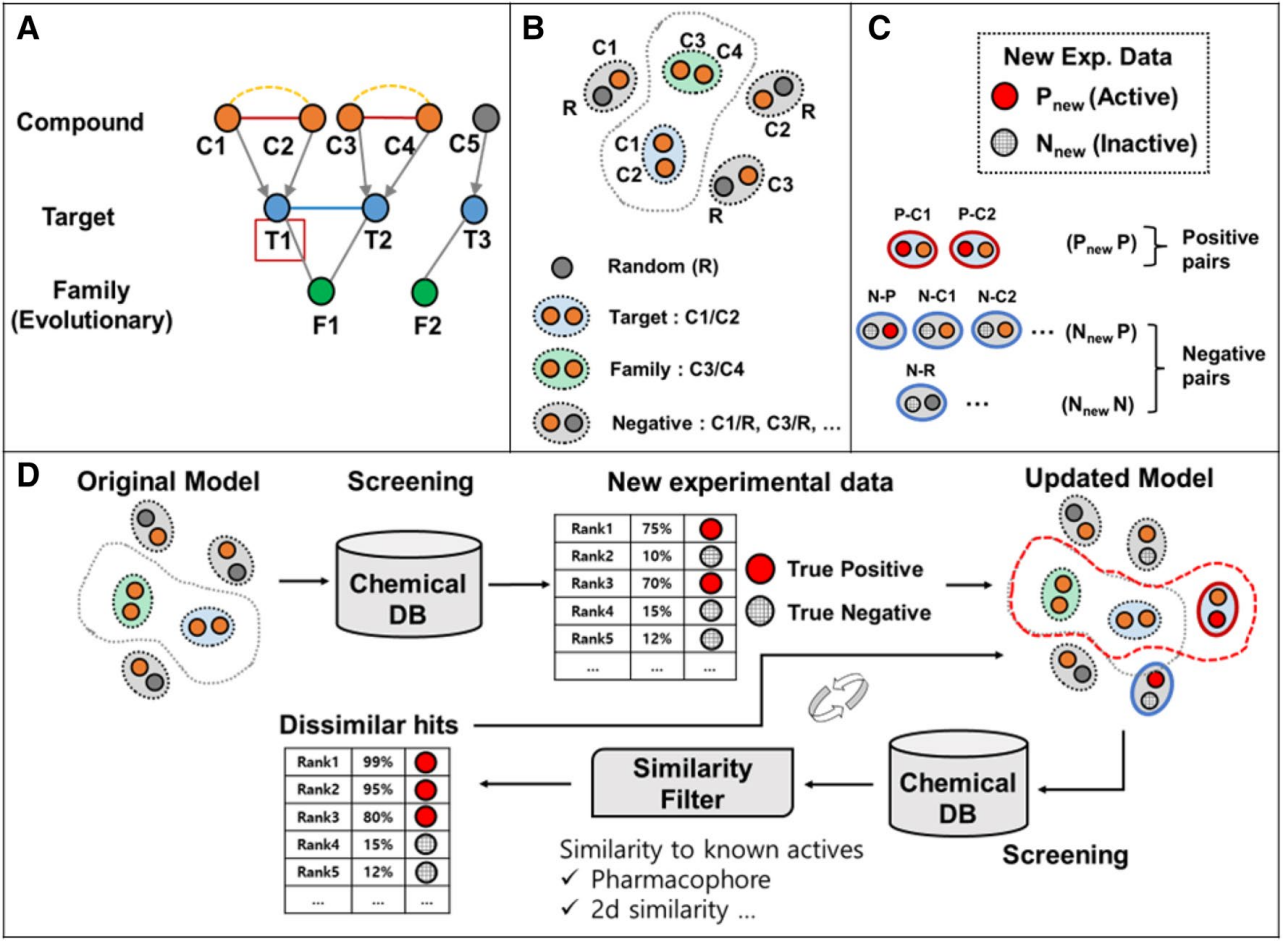
Schematic representation of ECBS retraining procedure with new chemical pair data. **A** Simplified example representing evolutionary relationships of chemicals defined by their targets and family information. T1 is a target of interest. **B** Definition of positive and negative samples is shown with the chemical compounds in **A**. **C** Different types of new chemical pair data are shown with newly-identified active (P_new_) and inactive compounds (N_new_). **D** The overall iterative VS strategy is illustrated

Here, we present an efficient chemical screening strategy that iteratively optimizes an MEK1-specific ECBS model using newly generated experimental validation data (Fig. 1). Using this approach, three new MEK1-binding hit molecules were identified and compared with previously identified MEK1 inhibitors to verify their structural novelty. Binding affinity was also determined for MEK2 and MEK5 to reveal the binding specificity among different MEK isoforms. Of the three, ZINC5814210 was found to be the most effective inhibitor of MEK1, MEK2, and MEK5, with sub-micromolar affinity in the range Kd 0.12–1.75 μM. To broaden the chemical space of MEK inhibitors, we used ZINC5814210 as a reference structure to generate new drug-like molecules with improved multiple binding statistics. Based on the calculated binding scores, several of these molecules have been suggested as potential MEK1 inhibitors with better binding affinities. Taken together, our results demonstrated the potential of an iterative ECBS-based screening approach for identifying novel hit molecules and optimizing their binding affinities.

## Results and discussion

### Iterative chemical binding similarity search to identify novel active compounds

Among the ECBS variants, the target-specific ensemble ECBS (TS-ensECBS) model is used to apply the chemical search strategy because of its flexibility which allows the inclusion of a variety of chemical relationships into a training set [6]. Generally, ECBS models categorize chemical pairs into two classes: evolutionarily related chemical pairs (ERCPs—positive data) and unrelated chemical pairs (negative data). ERCPs refer to chemical pairs that bind identical or evolutionarily related targets, such as the chemical pair C1–C2 or C3–C4 in Fig. 1A sharing the same target T1 or T2, and the chemical pairs C1–C3 or C2–C4 binding evolutionarily-related targets T1 and T2. Negative data, on the other hand, refers to chemical pairs that has no evolutionarily-related binding targets. Negative chemical pairs can be generated by pairing previously identified active compounds with randomly selected unrelated compounds (Fig. 1B). Random compounds can serve as useful negative training data for initial VS, which aims to retrieve potential hits from a large pool of unrelated chemical compounds. However, the performance of this screening on a set of structurally related chemical compounds with minor chemical modifications may be inadequate because of insufficient true-negative data. To enhance the model performance, we assumed that incorporating failed prediction data (false positives) as well as newly identified active compounds (true positives) contributes to refining the searchable chemical space of the original model and thus improves its prediction accuracy.

Three types of chemical-pairing schemes were devised to integrate the initial experimental validation data into the chemical similarity model. New inactive compounds (false positives by an initial prediction model) were paired with known active compounds (negative–positive, NP) or randomly selected negative compounds (negative–negative, NN) (Fig. 1C). In contrast, new active compounds (true positives by an initial prediction model) were paired with known active compounds (positive–positive, PP) or new inactive compounds (positive–negative, PN). For training ECBS, both NP (or PN) and NN chemical pairs were considered negative samples because of the absence of common or evolutionarily related binding targets, whereas PP chemical pairs were considered positive because of shared target-binding activity.

The original ECBS prediction model was retrained by incorporating different combinations of the new chemical-pair data (Fig. 1D). The retrained ECBS model with the highest prediction accuracy was used to search the chemical library again to identify new secondary hit molecules. To prioritize novel chemical scaffolds and ensure drug developability, chemical similarity filters and clustering approaches were used to select structurally novel and minimal scaffolds that could be distinguished from previously known active compounds (Fig. 1D).

### Comparison of screening performance with different types of chemical pairing data

The screening performances of the retrained ECBS models were assessed for different combinations of chemical-pairing data generated from the newly discovered active (P_new_) and inactive chemical compounds (N_new_). Each type of new chemical pair data (PP, NP, and NN) was first evaluated to estimate its individual impact on screening accuracy.

In general, the test results for the four targets (MEK1, WEE1, EPHB4, and TYR) indicated that the NP data considerably improved model performance, whereas PP and NN had minor effects (Table 1). This suggested that the inclusion of negative chemical pair data (NP) based on new inactive compounds greatly contributes to improving the model performance by providing true negative data. Models built using only unrelated random compounds will likely produce rough decision boundaries, thereby hindering the sensitive detection of minor activity-related chemical changes. Despite the marginal improvement, positive chemical pairs (PP) are expected to expand the chemical search space of ECBS models if active compounds with structurally novel chemical scaffolds are detected in the future. Generating negative data with random compounds (NN) also showed considerable model improvement, probably due to the effect of including true negative data, similar to NP. The increase in prediction accuracy was more if combinations of chemical pair data were used (Table 1 and Additional file 1: Table S1). The PP–NP or PP–NP–NN combinations showed the highest accuracy owing to their complementarity. When the prediction performance of the ECBS model (using PP–NP–NN) was compared with that of the standard single-chemical-based model, the ECBS model showed comparable or slightly better performance than the standard model (Additional file 1: Table S2).

**Table 1:** Comparison of model performance for the four test targets using different chemical pairing scheme

	WEE1 (P30291)	MEK1 (Q02750)	EPHB4 (P54760)	TYR (P14679)	Avg. AUC
Number of chemical data					
Known actives (< 100 nM)	19	24	22	22	
New exp. data (active/inactive^a^)	0/31	4/23	1/30	3/17	
AUC PR					
None	0.736	0.795	0.681	0.612	0.706
P_new_P_prv_ (PP)	0.744	0.758	0.669	0.690	0.715
N_new_P_prv_ (NP)	0.832	0.809	0.746	0.651	0.760
N_new_N_prv_ (NN)	0.757	0.821	0.695	0.651	0.731
PP–NP	0.828	0.803	0.743	0.748	0.781
NP–NN	0.839	0.829	0.753	0.665	0.772
PP–NN	0.762	0.773	0.693	0.710	0.735
PP–NP–NN	0.839	0.815	0.745	0.742	0.785

Experimental chemical activity data and cross-validation results (AUC of Precision-Recall curve) are shown for each target. The chemical compounds are labeled as P_new_ (new active), P_prv_ (previous active), N_new_ (new inactive), and N_prv_ (previous random inactive data)

^a^Chemical compounds with lower than POC 20% and higher than 80% are defined as active and inactive compounds, respectively

### Effect of chemical pairing data in MEK1

Regardless of these general trends, the impact of different chemical pairing data on the prediction performance varied for each test case. For example, NN and NP were much more effective than PP in MEK1, whereas PP contributed the most in TYR. The importance of including NN data in MEK1 suggests that including new inactive compounds and their relationships with random negative data may be more important than including new positive data.

Similarity learning involves training the distances between samples such that similar samples are close, but dissimilar samples are far from each other. Thus, retraining with NN will adjust the distances between the new inactive and random compounds; negative compounds (both inactive and random) are more effectively represented by maintaining the distances between active and inactive compounds and by providing information that inactive compounds are not similar to each other. We presumed that adjusting these relationships for negative data contributes to the increased predictive performance in MEK1. In contrast, PP had little impact on the predictive performance probably due to insufficient data around the new active compounds. We hypothesized that the potential false positives generated by including new active compounds into a training set may be the reason for the inefficiency of PP. Nevertheless, to ensure reliable conclusions, it is essential to perform large-scale analyses with a sufficient number of test cases in the follow-up study.

### Protein–ligand binding and cell viability assay for MEK1 inhibitors

The ECBS model, retrained with the new PP–NP chemical pair data, was used to screen MEK1 inhibitors from a virtual chemical library because it showed high accuracy and required less training data. The ECBS model with PP–NP was considered more suitable for a general VS task because PP–NP showed almost identical performance to PP–NP–NN with less training data (Table 1). According to our estimation, NN has approximately four times larger data size than PP or NP (Additional file 1: Table S3).

**Fig. 2:**
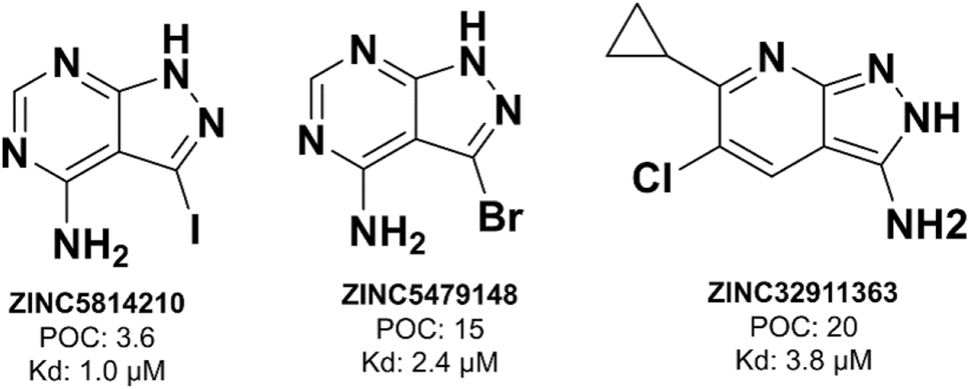
Two-dimensional structures of the new MEK1 inhibitors (ZINC5814210, ZINC5479148, and ZINC32911363), experimentally proven through scanELECT and KdELECT services from DiscoverX. The POC at 10 μM and Kd values are annotated (the lower values of POC and Kd indicate higher binding affinities)

Using the retrained ECBS model, 20,467 molecules were retrieved from the drug-like subset of the ZINC database, with a score cutoff of 0.8. Among them, 153 molecules were selected by excluding MEK1 pharmacophore-matched or structurally similar molecules to known active compounds. The candidates were finally narrowed to 17 by performing molecular clustering (i.e., selecting cluster centers). The results of the competitive binding assay revealed three strong MEK1 binders (Fig. 2) with percent of control (POC) values below 25% at 10 μM, with ZINC5814210 exhibiting the highest inhibitory activity (POC = 3.6%) (Table 2 and Additional file 2 : Table S1). A lower POC represents higher competitive binding to MEK1. Thermodynamic dissociation constant (Kd) of the three MEK1 binders was subsequently determined to be 1.0 µM (POC 3.6%) for ZINC5814210, 2.4 µM (POC 15%) for ZINC5479148, and 3.85 µM (POC 20%) for ZINC32911363, respectively. The absence of a plateau in the dose–response curves for some chemicals like ZINC32911363 could indicate unfavorable drug-like properties (such as weak affinity, low chemical stability, and limited cell permeability), making them less suitable candidates for subsequent optimization (Additional file 1: Figure S1).

**Table 2:** The percentage of control (POC) values for the 17 molecules tested at 10 µM using Protein–Ligand binding assay (scanELECT by DiscoverX)

Name	POC (%)	CAS Registry Number
ZINC5814210	3.6	151266-23-8
ZINC5479148	15	83255-86-1
ZINC32911363	20	1135283-22-5
ZINC4023558	43	42951-65-5
ZINC48325380	72	1223037-63-5
ZINC261507424	77	1640120-88-2
ZINC32581883	85	1125427-49-7
ZINC72156820	88	1360224-51-6
ZINC0130101	90	329712-63-2
ZINC16052626	91	848635-49-4
ZINC1573667	100	2993-05-07
ZINC70450773	100	1338218-78-2
ZINC84199299	100	1378862-01-1
ZINC8557584	100	93352-69-3
ZINC81947236	100	946157-08-0
ZINC0061493	100	2550–73-4
ZINC9419630	100	121371-16-2

**Fig. 3:**
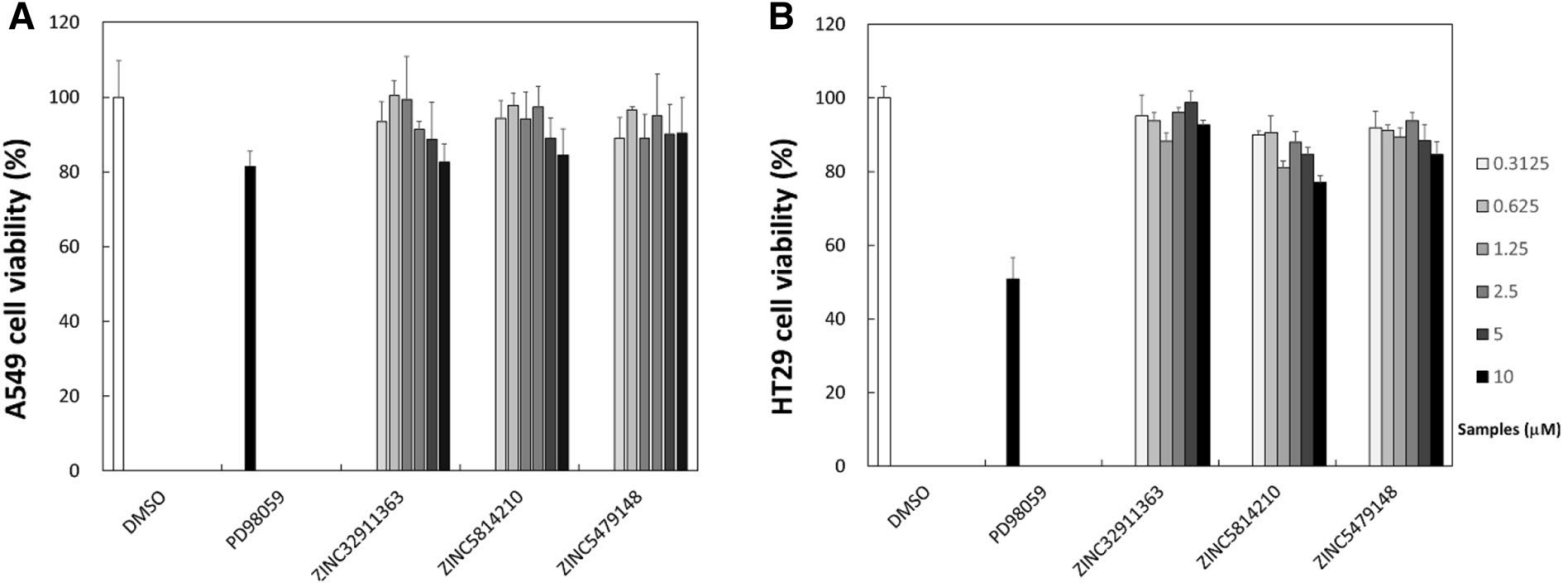
Results of cell viability assay with **A** A549 and **B** HT29 cell lines. To evaluate the cellular efficacy of the compounds against cancer cells, A549 lung cancer and HT29 colon cancer cells were selected to observe the relation between the cancer cell growth inhibition and MEK inhibition. PD98059 is a known selective cell permeable inhibitor for MEK1 (IC50 = 2 ~ 7 µM) and MEK2 (IC50 = 50 µM) and was used as a positive control in the present study. The two MEK inhibitors (ZINC5814210 and ZINC32911363) showed activity against A549 and HT29 cancer cells, especially at higher concentrations

Cell viability assays were performed to evaluate the anticancer activity of the MEK1 inhibitors. It showed that ZINC5814210 and ZINC32911363 had comparable activity to PD98059, a known selective cell permeable MEK inhibitor (IC50 2–50 µM) (Fig. 3). Especially, in the A549 cell, PD98059 showed about 80% cell viability at 10 μM which was similar to that of ZINC5814210 and ZINC32911363 (Fig. 3A). This also suggests that MEK inhibition alone has moderate cellular activity and requires additional effects to inhibit cell growth completely [18].

Notably, ZINC32911363 and ZINC5814210 showed similar activity in the A549 cell line, despite the weak biochemical activity of ZINC32911363 to MEKs. The discrepancy between biochemical and cell-based assay outcomes has been a recurring challenge when the new inhibitor identified from the biochemical studies is applied to cell-based studies. Because this discrepancy has been ascribed to the low cell permeability of the compound, we have attempted to elucidate the relationship by calculating logP values, which are indicative of compound cell permeability (Additional file 1: Table S4). The result showed that all the three compounds were within the moderate logP range (0.62 < LogP < 1.83), showing a drug-like property. Therefore, we guess that other factors may also contribute to variations in cell-based activity such as compound insolubility, nonspecific binding with serum proteins in the culture medium, interactions with culture plates, metabolic transformation, potential off-target effects, and pathway cross-talk [19]. Since the compounds have not yet been optimized, improving their anti-proliferative activity by conducting lead optimization and investigating the cell-specific variations in anti-cancer activity would be an interesting future work.

### Structural novelty of the new MEK inhibitors

Through ECBS retraining and chemical structural filtering, the newly discovered MEK1 inhibitors were found to have low structural similarity to known MEK1 inhibitors. In addition, three different similarity criteria were used to verify the structural novelty: molecular clustering, 2D fingerprint similarity, and substructure searching.

#### Molecular clustering

**Fig. 4:**
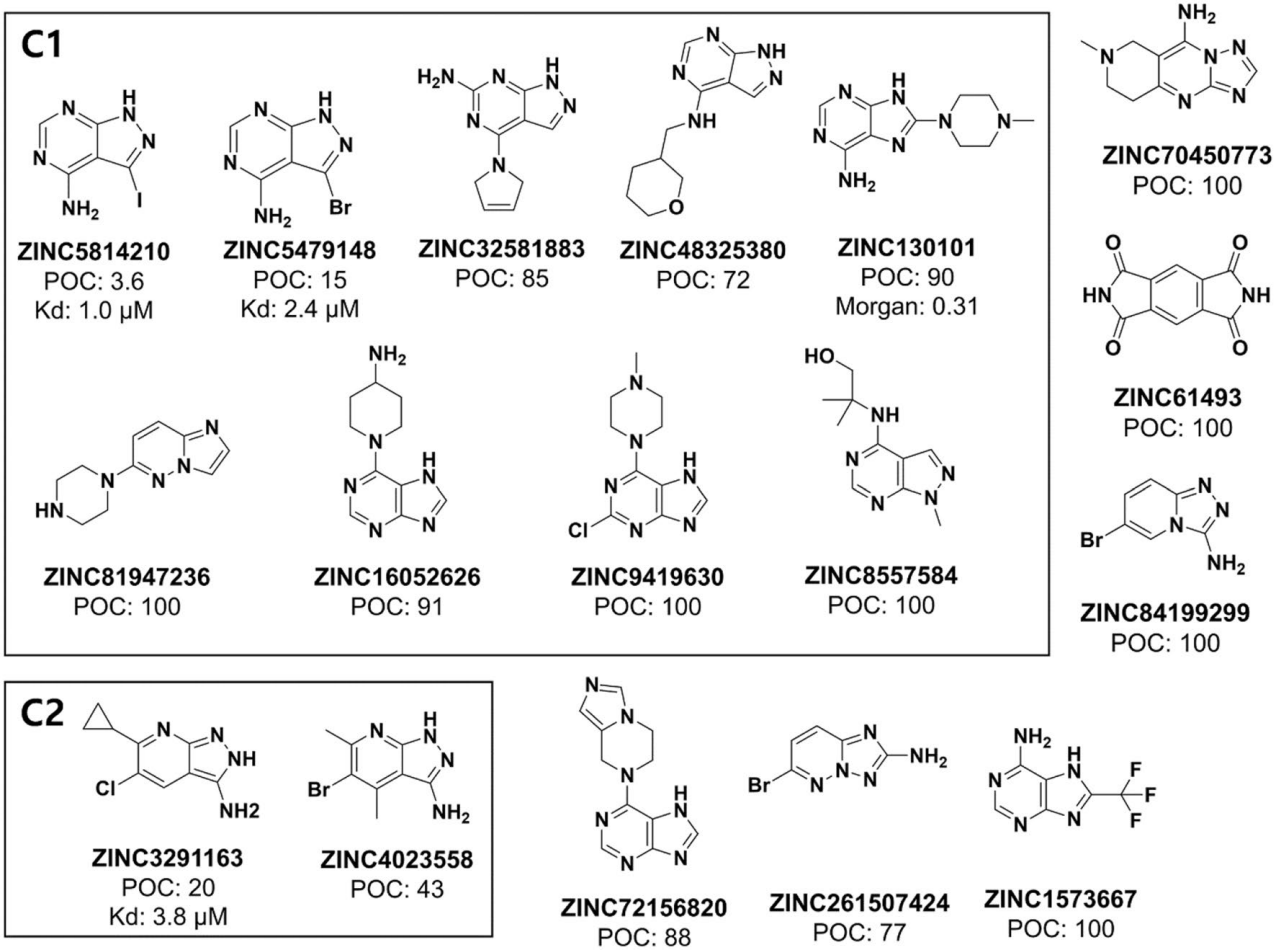
Molecular clustering results of all tested molecules for MEK1. The compounds (six weak or non-binders) not in C1 or C2 are not clustered

The 17 experimentally tested molecules were clustered using the RDK5 subgraph-based fingerprint (FP) to determine structure activity relationship (SAR) within each cluster. Eight clusters were formed when the similarity cut-off (Tanimoto coefficient) was set to 0.6. In the first cluster (C1 in Fig. 4), ZINC5814210 and ZINC5479148 clustered with seven very weak binders or non-binders. The second cluster (C2 in Fig. 4) consisted of another new MEK1 inhibitor, ZINC32911363, and a weak MEK1 binder, ZINC4023558 (POC 43%). The remaining clusters comprised six weak binders and non-binders. The four molecules in C1 (ZINC5479148, ZINC32581883, ZINC48325380, and ZINC130101) had a core structure similar to that of ZINC5814210; however, their MEK1 inhibition was much weaker. A comparison of ZINC5814210 with ZINC5479148 (or other similar molecules in C1) suggested that the presence of iodine or any halogen atom at the 10th atomic position in ZINC5814210 may be crucial for MEK1 inhibition, which was not found in previously known MEK1 inhibitors.

#### 2D fingerprint similarity

**Fig. 5:**
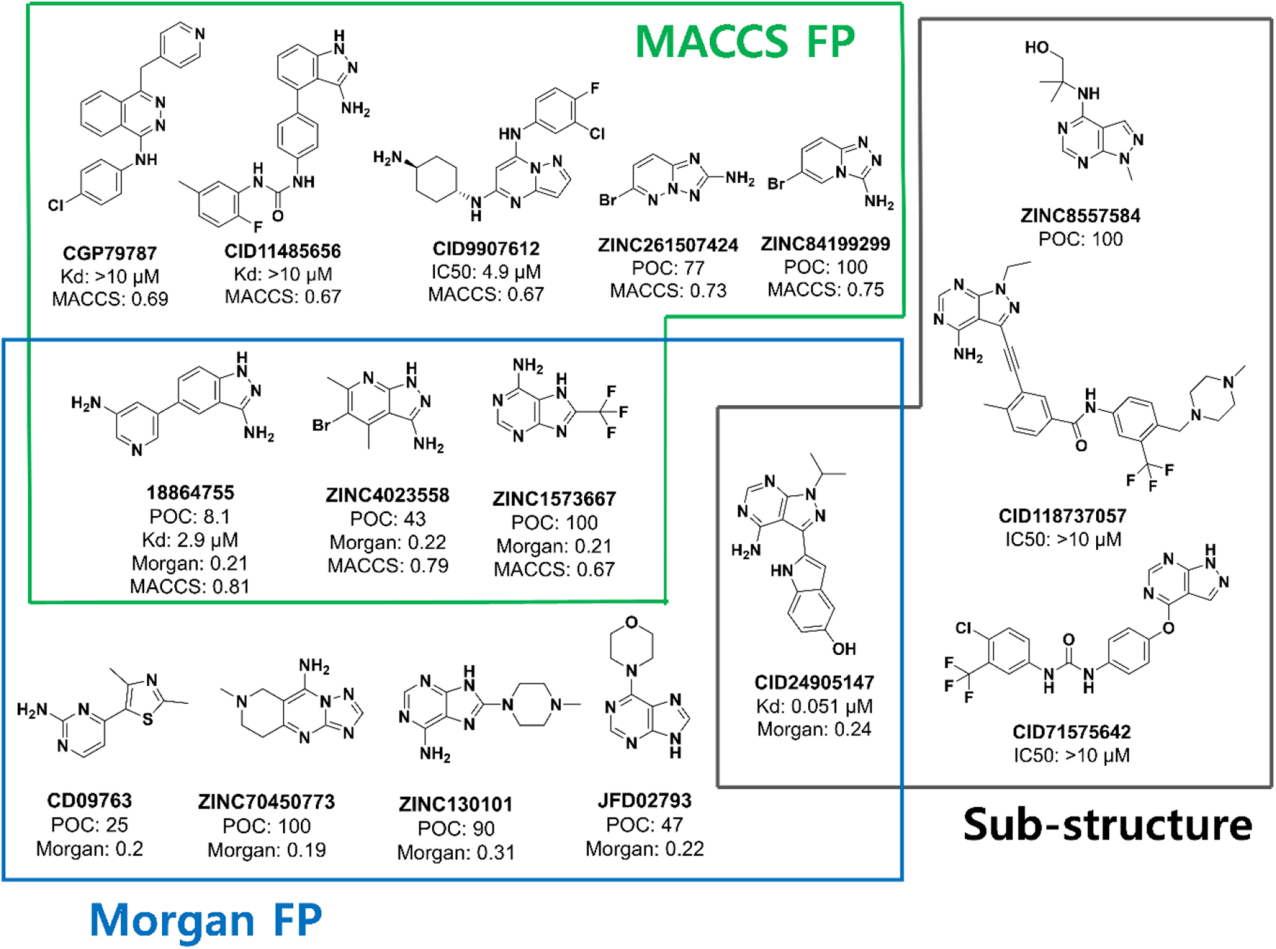
Molecules that are structurally similar to ZINC5814210 were identified using multiple structural criteria, as shown by green (MACCS fingerprints), blue (Morgan fingerprints), and gray (Substructure search) box. Tanimoto Coefficient (TC) was used to define molecular similarity with MACCS or Morgan fingerprints. The known experimental affinity values (POC, IC50, or Kd) are annotated with similarity scores

Additional 2D chemical similarities (Tanimoto coefficient) were calculated using Morgan and MACCS key 2D fingerprints for comparison with known MEK inhibitors, and the molecules most similar to ZINC5814210 were manually inspected (Fig. 5). The similarity calculation included 17 molecules tested in this study, 33 molecules from our previous study [8], and 692 MEK1-related molecules (486 molecules with IC50, Kd, or Ki values less than 10 µM) from BindingDB. ZINC5479148 (Kd 2.4 μM) and ZINC32911363 (Kd 3.85 μM) found in this study were excluded from the similarity search results.

Both fingerprints identified three common similar molecules; 18864755 (POC 8.1%, Kd 2.9 μM), ZINC4023558 (POC 25%), and ZINC1573667 (POC 100%) (Fig. 5). Among the three, 18864755 and ZINC4023558 were part of our previous study [8] whose chemical information had already been used to retrain the ECBS model, whereas ZINC1573667, validated in the present study, was also retrieved by both fingerprints. A strong MEK1 inhibitor, CID24905147 (Kd 51 nM from BindingDB), was identified using Morgan fingerprints with a similarity score of 0.24; however, it possessed bulky aromatic rings and alkyl groups. In contrast, MACCS fingerprints revealed that CID9907612 (IC50 4.9 μM from BindingDB) had a score of 0.67, but its chemical constituents were quite different from those of ZINC5814210. The low similarity scores and distinct chemical substituents in these molecules revealed that ZINC5814210 could be an effective hit molecule with a minimally active scaffold. All the remaining molecules retrieved by 2D fingerprints were weak MEK1 binders from our own studies or BindingDB.

#### Sub-structure similarity

The substructures of ZINC5814210 were compared to compounds examined with MEK1 using Discovery Studio Client (DSC) v19.1, ignoring the amino and iodo groups. CID24905147, listed by MACCS fingerprints, was also identified, but the others were all MEK1 non-binders (ZINC8557584 from the present study and CID118737057 and CID71575642 from BindingDB) (Fig. 5). No molecules with the same substructure as ZINC5814210 and with MEK1 binding properties were found in the Reaxys database (Additional file 1: Figure S2).

### Cross-activity to the MEK isoforms and docking models

MEK1/2 and MEK5 are targeted by drugs because of their additive effects [14]; however, limited information exists regarding their binding specificity and promiscuity. We tested three MEK1 inhibitors on MEK2 and MEK5, which share 86% and 44% sequence similarity with MEK1 [20]. The results showed that ZINC5814210 had the strongest inhibitory effect on all MEK isoforms, with eight times higher inhibition of MEK5 than MEK1 and 15 times higher inhibition of MEK5 than MEK2 (Table 3). ZINC5479148 and ZINC5814210 showed similar inhibition of MEK1 and MEK2 and higher inhibition of MEK5 than MEK1/2. In contrast, ZINC32911363 showed low inhibition of MEK2 despite the high sequence similarity between MEK1 and MEK2.

**Table 3:** Cross inhibitory activity (Kd, nM) of the three MEK1 inhibitors to the MEK isoforms

Compound Name	MEK1	MEK2	MEK5
ZINC5814210	1000	1750	120
ZINC5479148	2400	5350^a^	235
ZINC32911363	3850^a^	> 10,000^a^	3950^a^

^a^Higher chemical concentration is required to reach a plateau and make more accurate Kd determination

**Fig. 6:**
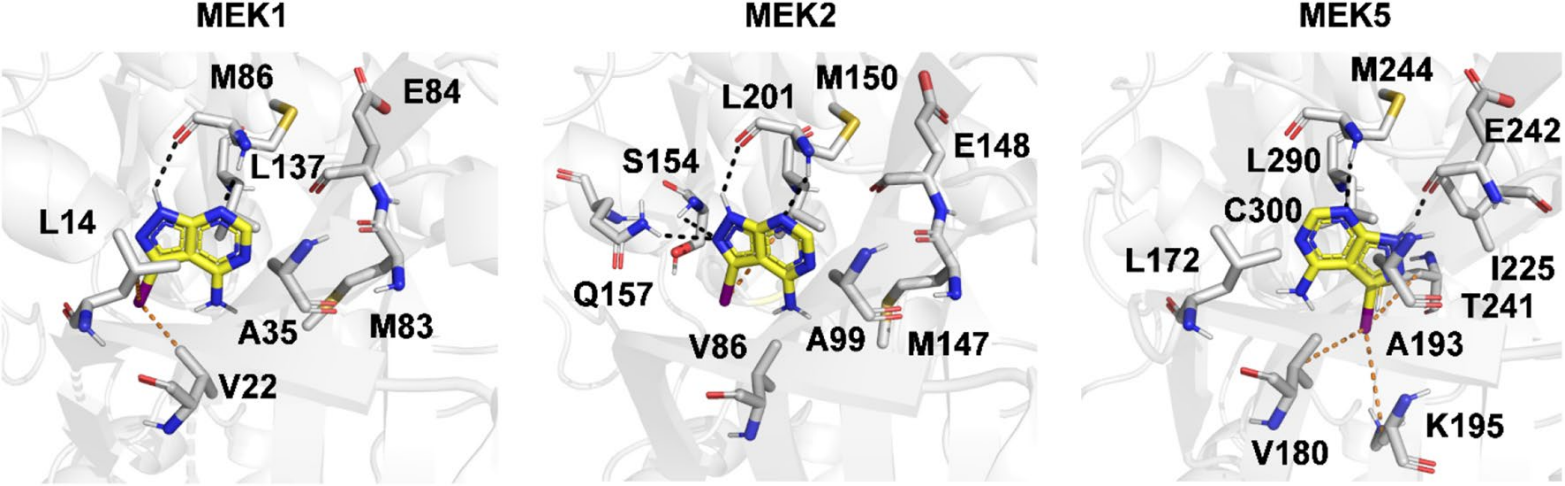
Molecular Docking results of ZINC5814210 for MEK1, MEK2, and MEK5. The carbon atoms of ZINC5814210 are shown as yellow sticks. The carbon atoms of residues that participate in interaction with ZINC5814210 and contribute to the hydrophobic environment around the ligand are shown as white sticks. The secondary structures of proteins are shown as cartoon. Hydrogen bond interactions and hydrophobic interactions are represented by black and orange dotted lines, respectively. X-ray crystal structures of human MEK1 and MEK2 (PDB accession codes: 3V01 and 1S9I) and Alphafold predicted structure of MEK5 were used for docking

Because the compounds showed unintended binding affinities for MEK5, we checked the binding targets of 24 known MEK1 binding compounds used for training (10 from DrugBank and 14 from BindingDB). However, out of 24, only one compound (BDBM50386693) from BindingDB had an IC50 value for MEK5, but its binding affinity was very low (IC50 > 10,000 nM) compared to IC50 2100 nM for MEK1. One hypothesis for the stronger MEK5 binding of ZINC32911363 is that non-optimized MEK1 inhibitors may have similar or higher binding affinities for MEK5 because of their similar binding pockets (Fig. 6 and Additional file 1: Figure S3). During lead optimization, the binding specificity to a particular MEK isoform likely increases with minimal cross-activity. Additionally, because the newly identified active compounds share a similar core structure, this particular type of chemical structure might have a higher binding affinity for MEK5.

To understand the molecular mechanisms underlying the dual inhibition of MEK1/2 and MEK5, we performed docking simulations with ZINC5814210 and compared the potential binding modes of the different MEK isoforms. The docking results suggested that the iodine atom of ZINC5814210 is crucial for interacting with the hydrophobic cavities of MEK1/2 and MEK5, leading to different inhibitory activities (Fig. 6). In particular, Val180, Lys195, and Cys300 in MEK5 formed extensive hydrophobic contacts with the iodine atom of ZINC5814210, whereas Leu14 and Val22 in MEK1 formed similar contacts. ZINC5814210 also formed hydrogen bonds with the backbone atoms of Met244 in MEK5 and Met86 in MEK1; the additional hydrogen bond with Glu242 in MEK5 may result in a higher binding affinity to MEK5 than to MEK1. The different binding orientations of ZINC5814210 between MEK1 and MEK5 may be due to the change from Thr241 (MEK5) to Met83 (MEK1), a small-to-large mutation in the ligand-binding pocket. In MEK2, the ligand orientation was similar to that in MEK1 and the hydrogen bond interaction with Met150 was conserved, as in the other two MEKs. The binding orientation of ZINC5814210 partially overlaps with that of ATP of MEK1 (PDB ID:3V01), even though there is not a perfect alignment between their adenine parts (Additional file 1: Figure S3). The distinct functional groups of ZINC5814210 notably lacking a phosphate group likely contribute to the different docking conformation to ATP. Nonetheless, the aromatic rings of ZINC5814210 and ATP are aligned, indicating partially conserved interactions around the adenine part.

### Chemical features for binding to MEKs

The structure activity relationship (SAR) study of the tested molecules (Figs. 4 and 5) reveals that halogen atoms are present in all three new MEK1 inhibitors. The core structure of ZINC5814210 was shared by three molecules (ZINC32581883, ZINC48325380, and ZINC130101 in the C1 cluster of Fig. 3), further highlighting the importance of halogen atoms in MEK1 inhibition. The second-best MEK1 binder, ZINC5479148, contained bromine instead of iodine in a similar position, leading to a two-fold reduction in MEK1 inhibition (from 1.0 to 2.4 μM). Similarly, replacing iodine with chlorine in ZINC32911363 resulted in a lower inhibitory activity (from 1.0 to 3.85 μM). These observations suggest that the hydrophobic contribution to MEK binding is enhanced in the presence of iodine but much less when bromine or chlorine is present in the ligand.

Because none of the docking poses of the three identified inhibitors in the MEK isoforms showed halogen bond interactions (Fig. 6), we focused on the hydrophobic interactions surrounding the iodine atom. Although halogen bonds are often given greater importance in studies on halogenated ligands, hydrophobic interactions are frequently overlooked [21]. However, recently, there have been implications for a recurring hydrophobic environment surrounding halogen atoms in the context of protein–ligand interactions [22]. Shinada et al. suggested that fluorine, bromine, and iodine exhibited similar tendencies to participate in carbon interactions, with percentages of 72.3%, 72.6%, and 71.3%, respectively. Heavier halogens are more frequently found in hydrophobic-rich environments (a minimum of four hydrophobic partners), with 6.8% in chlorine and 13.4% in iodine [22].

In contrast, the previously reported MEK1 inhibitors CID24905147 and 18864755, which were similar to ZINC5814210 in our study, were not halogenated (Fig. 5). Therefore, they inhibited MEK1 by non-bonded interactions without halogen atoms. The docking results were similar to those of ZINC5814210 in MEK1 as they shared common interactions with Val22, Glu84, and Met86 (Additional file 1: Figure S4). Thus, we propose that a combination of hydrophobic interactions provided by the iodine atom in ZINC5814210 and a few hydrogen bonds contribute to its strong binding affinity for MEK1, MEK2, and MEK5. The docking poses of ZINC5479148 and ZINC32911363 in the three MEKs also supported the idea that hydrophobic and hydrogen bond interactions are crucial for MEK inhibition (Additional file 1: Figures S5 and S6).

### Generation of chemical derivatives starting from ZINC5814210

**Fig. 7:**
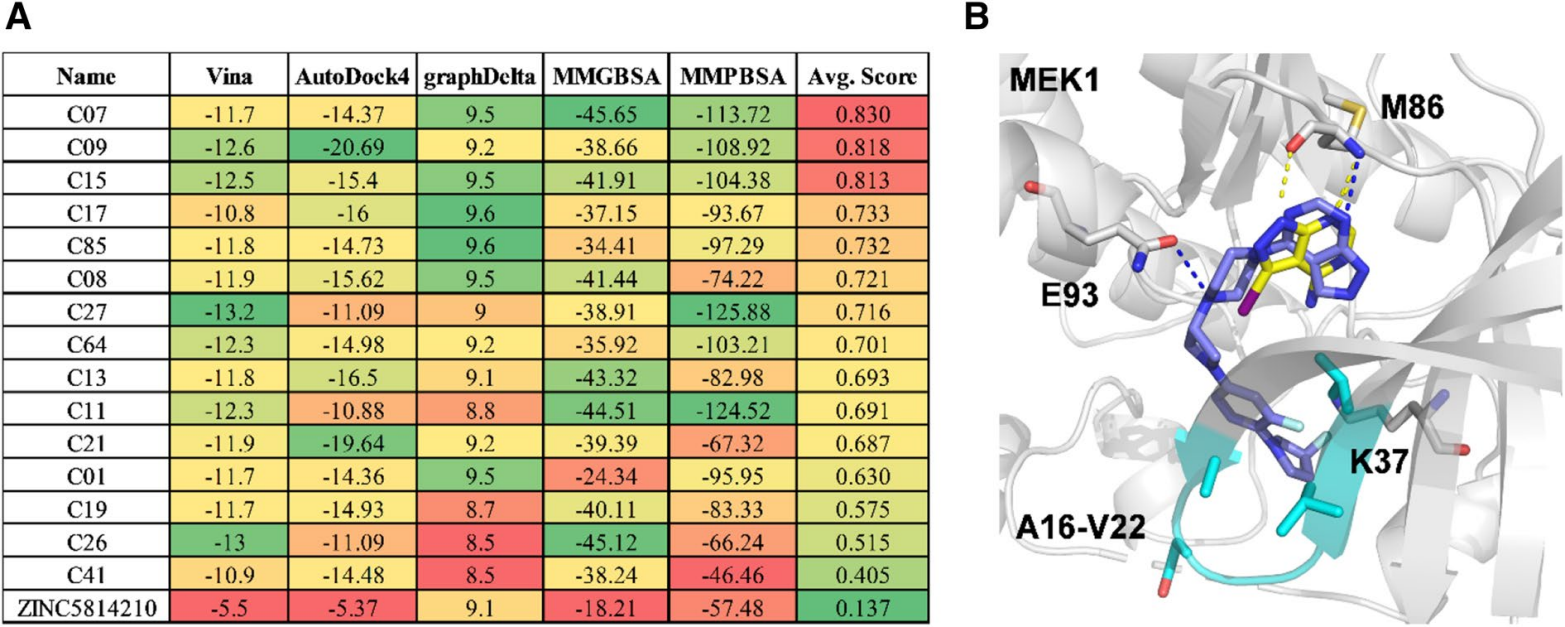
Different binding scores for designed molecules generated from ZINC5814210. **A** The docking conformation of 15 designed molecules were evaluated using different scoring methods (including Autodock Vina, AutoDock4, graphDelta, MM/GBSA, and MM/PBSA). Each score was min–max normalized (0–1) and averaged (Avg. Score). **B** Molecular Docking result of C07 in MEK1. The carbon atoms of C07 are shown in blue and the beta sheet region (Ala16-Val22, and Lys37) that contacts with C07 is shown in cyan. The X-ray crystal structure of human MEK1 (PDB ID: 3V01) was used for docking

The dual binding ability of ZINC5814210 to MEK1/2 and MEK5, and its low molecular weight make it a desirable starting point for the development of stronger derivatives. To verify this possibility, using the REINVENT [23] and DOCKSTREAM [24] methods, we generated 200 diverse drug-like molecules with improved docking scores based on the docking complex structure of ZINC5814210 and MEK1 (Fig. 6). This computational chemical generation experiment was based on the assumption that structure-based methods are generally acceptable for estimating ligand-binding affinity [25, 26]. When we checked the convolution neural network (CNN) affinity scores of GNINA to MEKs, ZINC5814210 and ZINC5479148 showed higher MEK5 binding scores than MEK1, which was consistent with the experimental data, although precise predictions for ZINC32911363 and MEK2 were not made (Additional file 1: Table S5). The cyclopropane ring of ZINC32911363 likely contributes to increase chemical lipophilicity (Additional file 1: Table S4), which might be responsible for the overestimated docking scores of ZINC32911363 despite the weak biochemical MEK-binding affinity. To avoid possible bias resulting from the use of one particular method, multiple structure-based methods, such as molecular docking, MM/PBSA, and MM/GBSA, were used to find common high-scoring candidates (Fig. 7A).

The generated molecules showed drug-like properties, with synthetic accessibility scores (by RDkit) ranging from 3 to 5, where 1 was easy to synthesize and 10 was the hardest. Of these, 15 were selected based on average scores from AutoDock Vina, AutoDock4.2, graphDelta, MM/GBSA, and MM/PBSA (Fig. 7A). The representative docking conformation using C07 with the highest binding score suggested similar chemical interactions to ZINC5814210, but with a larger chemical group replacing the iodine atom (Fig. 7B). In contrast to ZINC5814210, C07 showed additional favorable interactions with the beta sheet region (Ala16-Val22, and Lys37) and Lys37 in MEK1. Nevertheless, experimental validation should be conducted to confirm the activities of these newly generated molecules.

## Conclusion

This study examined the optimization of the ECBS model by incorporating the initial VS validation data on MEK1. A refined ECBS model was used to identify novel hit molecules for MEK1. The most effective inhibitor, ZINC5814210, showed little structural similarity to previously known MEK1 inhibitors as determined by multiple chemical structure similarity searches and pharmacophore analyses. Using molecular docking models, we also assessed the binding activity between ZINC5814210 and MEK isoforms (MEK2 and MEK5), which suggested relevant structural features for MEK-binding. Further chemical novelty was explored by predicting ZINC5814210's binding targets using the Structure Ensemble Approach (SEA) [27] and SwissTargetPrediction [28] methods; however, no MEK-related targets were found (Additional file 1: Tables S6 and S7). The potential to improve MEK binding was further examined through computational molecular generation, which suggested the possibility of designing more promising active compounds. Iterative chemical synthesis, experimental validation, and model retraining with these compounds will aid in further improving the prediction model, as performed using the initial VS data. This approach demonstrates the advantages of using ECBS in conjunction with generative modelling to design molecules. To introduce additional therapeutic options, this strategy can be evaluated using several other targets.

## Methods

### Training and testing evolutionary chemical binding similarity with new experimental data

The ECBS model is based on a binary classification (classification similarity learning) model; the training set consists of positive and negative chemical-pair samples. Positive data refer to evolutionarily related chemical pairs (ERCPs) that bind to common or homologous targets, while negative data represent chemical pairs that are not ERCPs. Negative data can be generated by chemical pairing of active and randomly selected (most likely inactive) compounds. The target-ECBS model confines ERCPs as chemical pairs that bind to common targets, whereas the family-ECBS model expands ERCPs as chemical pairs that bind to any evolutionarily related targets. The ensemble ECBS (ensECBS) model integrates these different evolutionary ECBS models (*X*-ECBS), each of which is defined using heterogeneous protein family databases, such as PFAM, SMART, and SUPERFAM. The target-specific ensECBS (TS-ensECBS) model was designed to accelerate the target-specific similarity search of the ensECBS model by confining the ERCPs related to a specific target. Details about the ECBS model training and scoring can be found in a previous study [6].

In a previous VS study [6], a TS-ensECBS model was created for MEK1 (UniProt ID Q02750) using chemical data from BindingDB (threshold 10 μM) and DrugBank, which comprised 27,906 positive and 167,436 negative chemical pairs generated from 860 evolutionarily related compounds (including 24 MEK1 inhibitors) and 5061 random compounds. The training data comprised 460 proteins homologous to MEK1 collected from the SMART, PFAM, and SUPFAM databases. Through initial screening using TS-ensECBS for MEK1, 6 active and 26 inactive compounds were successfully identified, and their 3D pharmacophore models for MEK1 binding were suggested [8]. The new 4 active and 19 inactive compounds were paired with chemicals from the original training set reduced with a binding affinity threshold 1 μM, which generated 645 PP, 528 NP, and 2667 NN chemical pair data. The new chemical-pair data were then combined with the original training data to retrain the ECBS model. The same procedure was applied to WEE1 (P30291), EPHB4 (P54760), and TYR (P14679) for which initial ECBS screening data were available [6].

The “ranger” Random Forest package in R was used for training and model building with default parameters. The retrained ECBS models with different combinations of chemical-pair data were evaluated using the area under the curve (AUC) value of the precision-recall (PR) curve (AUCPR) from sevenfold cross-validation. Because the AUCPR baseline performance varies according to the ratio of positive/negative labels in the test set, we generated common test data applicable to all ECBS models. Individual chemical compounds for each target were split into training and test sets for cross-validation, and all chemical pairs containing the selected test compounds were considered as a common test set to calculate the AUCPR values. By contrast, the remaining training data varied according to the model.

### Virtual screening with retrained ECBS model

For VS, a drug-like subset (661,339) from the ZINC database (downloaded on June 4th, 2020) was used as the chemical library. The chemical library was converted into binary fingerprints (MACCS and FP4 using Open Babel) to screen for MEK1 inhibitors using ECBS. FP4 is a substructure fingerprint based on SMARTS patterns and implemented in Open Babel. The retrained ECBS model with the highest AUCPR value (PP–NP in Table 1) was used to calculate similarity scores between the chemical library and known MEK1 inhibitors. The original and retrained ECBS models were identical, except that the latter included experimental data into a training set. The highest similarity score assigned to a chemical compound in the chemical library was used to select final candidates for subsequent experimental validation.

### Molecular filtering by 2D structure similarity and pharmacophore match

The chemical library was filtered based on a prebuilt pharmacophore model and chemical structure similarity with known MEK1 inhibitors. Pharmacophore models built for MEK1 [8] were applied using the Ligand Profiler protocol in DSC and all molecules with non-zero fit scores were excluded. Chemical structure similarity (Tanimoto coefficient) to previously-identified MEK1 inhibitors (from Binding DB with 10 μM cutoff) was calculated using FCFC_6 fingerprints in DSC, with the highest similarity score taken as representative. Molecules with a similarity score higher than 0.47 (p-value 0.01) were excluded. The remaining molecules were sorted by ECBS scores, and 153 molecules with a score above 0.8 were clustered with FCFP_6 fingerprints in DSC. The resulting 17 molecules (cluster centers) were used for the subsequent competitive binding assay. Clustering was performed to select the minimal and most conserved chemical core structures among the cluster members. Nonspecific binding of these molecules was checked using the PAINS filter in RDKit. Information on the binding targets, patents, and bioactivity data for the tested chemicals was manually checked using the Reaxys, ChEMBL, and PubChem databases to ensure chemical novelty before purchase.

### Competitive ligand binding assay

Chemical compounds were purchased from InterPharm (http://www.interpharmcorp.com) with a minimum purity of 90%. Experimental validation of chemical binding to MEKs was performed using the scanELECT and KdELECT services from DiscoverX [29]. In scanELECT, quantitative detection of kinase binding is accomplished by comparing the amount of kinase captured on a solid support in the presence of both test and control ligands. The kinase inhibition activity was estimated by evaluating the inhibitory percentage (%) of a control ligand (POC) at 10 μM. The 10 μM was used as a threshold to define active compounds. For molecules with less than 25% POC, binding dissociation constant (Kd) values were determined using KdELECT from DiscoverX by fitting to a standard dose–response curve using the Hill equation.

### Cell viability assay

A549 and HT29 cells was purchased from Korea cell line bank, and cultured in RPMI-1640 medium (22400-089, Thermo Fisher Scientific) supplemented with 10% (v/v) heat-inactivated FBS (12483020, Gibco), 100 U mL^−1^ of penicillin and 100 μg ml^−1^ of streptomycin (penicillin–streptomycin, 15140122, Thermo Fisher Scientific). Cells were maintained in a humidified incubator at 37 °C and 5% CO_2_.

Next, 10 mM DMSO stock solutions of PD98059 (positive control), ZINC5814210, ZINC5479148, and ZINC32911363 were diluted in the cell culture medium. The prepared compound solution was added to 96-well microplates (SPL) in triplicate, and serial 1/3 dilution were performed. The volume of each well was set to 50 μL. Cell culture medium containing 50,000 cells/mL was prepared in a reagent reservoir, and 50 μL of the prepared cell-containing medium was added to 96 a well micro plate (Corning) to seed approximately 1,000 cells/well. The plates were then incubated at 37 °C and 5% CO_2_ for 24 h. 50 μL of the prepared compound solution was added to each well of a cell plate, and the plate was incubated at 37 °C and 5% CO_2_ for 72 h. Cell viability was measured using the EZ-Cytox cell viability assay. Then 10 μL of EZ-cytox reagent was added to each well, and the plate was incubated at 37 °C and 5% CO_2_ for 1 h. UV absorption was recorded using a plate reader. Average cell viability was calculated using the following formula:

$${\text{Cell viability }}\left( {\text{\% }} \right) = 100{ } \times \frac{{\left( {{\text{OD}}\;{\text{of}}\;{\text{compound}}\;{\text{wells}} - {\text{OD}}\;{\text{of}}\;{\text{blank}}\;{\text{well}}} \right)}}{{\left( {{\text{OD}}\;{\text{of}}\;{\text{DMSO}}\;{\text{wells}} - {\text{OD}}\;{\text{of}}\;{\text{blank}}\;{\text{well}}} \right)}}$$

### Structural similarity of ZINC5814210 to known MEK1 inhibitors

The 33 MEK1 tested molecules in our previous study [8] and 692 MEK1 tested molecules (486 molecules with IC50, Kd, or Ki values less than 10 µM) from Binding DB [30] along with seven MEK1 binders from DrugBank [31] were collected for checking structural similarity with ZINC5814210. We performed a structural similarity analysis on ZINC5814210 using 2D fingerprints, substructure matching, and clustering approaches. The Tanimoto coefficient similarity was calculated using Morgan fingerprints (radius of 2, 1024 bits) and MACCS keys (166 bits fingerprint) in RDKit, in addition to the FCFC_6 fingerprints in DSC used as a similarity filter in the VS procedure. The “*Align to substructure*” protocol in DSC was used for substructure matching, and Reaxys database was used together to find molecules with the same substructure as ZINC5814210. Clustering was performed using the Butina algorithm in the RDKit. All the 2D chemical structures were obtained using ChemDraw 20.1.1. A structural analysis was performed following the OpenCADD tutorial [32].

### Protein structure modelling, and molecular docking to MEK isoforms

The X-ray crystal structures of human MEK1 (PDB accession code:3V01) and MEK2 (PDB accession code:1S9I) were obtained from the PDB database. In DSC, co-crystallized ligands and ions were removed and the resulting protein structure was prepared by building missing loops using a modeller, followed by energy minimization and protonation. The MEK5 structure was modelled using AlphaFold v2.1.0 [33].

Molecular docking was performed using GNINA 1.0 [34] with 1000 Monte Carlo chains. The XYZ dimensions were 40 Å and the center coordinates of XYZ were estimated based on the bound ATP. For the MEK5 structural model, ATP bound to MEK1 was grafted, and the center coordinates were used as a docking initiation site. A RMSD filter value of 1.0 Å was applied to eliminate multiple closely related binding poses, and the docking conformation with the best docking score was used for the structural analysis.

### Molecular design and generation from ZINC5814210

The REINVENT 3.0 tool [23] was trained using filtered ChEMBL [35] data for 20 epochs to create a new general agent. The pretrained agent was introduced into a set of known MEK inhibitors, including ZINC5814210 to obtain a focused agent. Consequently, 200 molecules were generated with multiple optimization conditions (i.e., molecule diversity, scoring functions based on better QED, molecular weight less than 550 Da, high similarity to ZINC5814210**,** and Autodock VINA [36] docking score between − 8 and − 12 kcal/mol) over 1000 epochs. The docking score was assigned a weight of 2 to prioritize high binding affinity, whereas other conditions were set to 1.

DockStream [24] integrated with REINVENT enables the incorporation of docking simulations into the molecule generative process, thus providing the agent with 3D structural information. To run DockStream, the MEK1 protein structure was prepared using PDBFixer, which added missing heavy atoms and hydrogen atoms at pH 7.4, built missing loops and standardized residues, and removed non-standard residues not relevant to structural modelling. The candidate ligands were input as the SMILES code, and conformer embedding was performed using the RDKit. The SMILES codes were standardized, and the ligands were protonated. The search space for docking was based on the ATP bound to the 3V01 protein. The grid size was set at 40 Å for the XYZ coordinates, and 1000 poses per ligand were generated. The docking procedure described in a previous study [37] was followed using Autodock4 [38] for cross-checking.

### Molecular dynamics and binding free energy calculations

The Molecular Dynamics (MD) simulations were conducted using Gromacs-2018 [39]. The CHARMM22 force field for the ligands were obtained using the SwissParam server [40]. The system was solvated using the SPC water model, and the system was neutralized. Periodic boundary conditions were applied in all directions, and energy minimization was performed using the steepest descent algorithm. The particle mesh Ewald method was used for long-range interactions with an electrostatic cutoff of 1.2 nm and van der Waals cutoff of 1.2 nm. The bond angles were restrained using the LINCS algorithm and the pressure was set to 1 atm using the Berendsen method. The temperature was regulated to 310 K using the V-rescale weak-coupling method. For position restraints in the NVT and NPT, 100 ps was set, followed by a 10 ns production run for each protein–ligand complex with a time step of 2 fs. The structural coordinates were saved every 1 ps, and final snapshots of the complexes were extracted using the GROMACS analysis tool. To calculate the endpoint binding free energies, the MM/PBSA and MM/GBSA methods were applied using the gmx_mmpbsa [41] and g_mmpbsa [42] tools, respectively. The binding free energy was computed using 100 snapshots sampled from the entire MD production run for each protein–ligand complex.

### Supplementary Information

**Additional file 1: Figure S1.** The duplicated dose response curves to determine Kd values of chemical compounds are shown for MEK1, MEK2, and MEK5. X-axis represents ligand concentration (nM) and Y-axis relative inhibitory activity by KdELECT service. **Figure S2.** Structurally similar molecules were identified via substructure search in Reaxys database. **Figure S3**. Molecular docking conformations of ZINC5814210 for MEK1, MEK2, and MEK5 are superimposed with ATP found in the MEK1 structure (PDB ID: 3V01). **Figure S4.** Two-dimensional interaction diagram of previously reported MEK1 inhibitors retrieved by 2D fingerprint similarity. **Figure S5.** Two-dimensional interaction diagram of MEK-ZINC5479148 docking models. **Figure S6.** Two-dimensional interaction diagram of MEK-ZINC32911363 docking models. Except MEK2, ZINC32911363 has better Kd binding affinity to other two MEKs. **Figure S7.** The molecules selected based on binding free energy scores from either MM/GBSA or MM/PBSA or both of the methods. **Table S1.** Experimental chemical activity data and cross-validation results (AUC of Precision-Recall curve) for each test target protein. **Table S2.** Comparison of the prediction performance of the standard single chemical-based Random Forest model with the ECBS model trained with PP-NP-NN data. **Table S3.** Estimation of chemical pair data size. **Table S4.** LogP values for the tested compounds. **Table S5.** GNINA docking scores for MEKs are shown with biochemical binding affinity data in Table 3. **Table S6.** The target prediction results for ZINC5814210 from Swiss target prediction server. **Table S7.** The target prediction results for ZINC5814210 from Structure Ensemble Approach (SEA) server.

**Additional file 2: Table S1.** SMILES for the tested compounds.

## Data Availability

The test data and code required to generate the results of this paper and run ECBS are available in the following GitHub repository: https://github.com/keunwan-kist/iter_ECBS_VS

## Abbreviations

ECBS: Evolutionary chemical binding similarity
TS-ensECBS: Target-specific ensemble ECBS
ERCP: Evolutionarily-related chemical pair
MEK1: Mitogen-activated protein kinase kinase 1
POC: Percentage of control
MD: Molecular dynamics
DSC: Discovery Studio Client
PR AUC: Area under the curve (AUC) of precision recall (PR) curve
PDB: Protein data bank
FP: Molecular fingerprint
SEA: Structure ensemble approach
