# Supplementary material for: Iterative machine learning-based chemical similarity search to identify novel chemical inhibitors
Source: J Cheminform. 2023 Sep 23;15:86. doi: 10.1186/s13321-023-00760-6 (PMC10517535; doi:10.1186/s13321-023-00760-6)
Supplement: Supplementary file 1 — Additional file 1: Figure S1. The duplicated dose response curves to determine Kd values of chemical compounds are shown for MEK1, MEK2, and MEK5. X-axis represents ligand concentration (nM) and Y-axis relative inhibitory activity by KdELECT service. Figure S2. Structurally similar molecules were identified via substructure search in Reaxys database. Figure S3. Molecular docking conformations of ZINC5814210 for MEK1, MEK2, and MEK5 are superimposed with ATP found in the MEK1 structure (PDB ID: 3V01). Figure S4. Two-dimensional interaction diagram of previously reported MEK1 inhibitors retrieved by 2D fingerprint similarity. Figure S5. Two-dimensional interaction diagram of MEK-ZINC5479148 docking models. Figure S6. Two-dimensional interaction diagram of MEK-ZINC32911363 docking models. Except MEK2, ZINC32911363 has better Kd binding affinity to other two MEKs. Figure S7. The molecules selected based on binding free energy scores from either MM/GBSA or MM/PBSA or both of the methods. Table S1. Experimental chemical activity data and cross-validation results (AUC of Precision-Recall curve) for each test target protein. Table S2. Comparison of the prediction performance of the standard single chemical-based Random Forest model with the ECBS model trained with PP-NP-NN data. Table S3. Estimation of chemical pair data size. Table S4. LogP values for the tested compounds. Table S5. GNINA docking scores for MEKs are shown with biochemical binding affinity data in Table 3. Table S6. The target prediction results for ZINC5814210 from Swiss target prediction server. Table S7. The target prediction results for ZINC5814210 from Structure Ensemble Approach (SEA) server. [file 13321_2023_760_MOESM1_ESM.pdf]

Additional file 1

Figure S1-S7

Table S1-S7

Iterative Machine Learning-Based Chemical Similarity Search to Identify Novel Chemical Inhibitors

Prasannavenkatesh Durai[1], Sue Jung Lee[2], Jae Wook Lee[2], Cheol-Ho Pan[1], Keunwan Park[1,3]*

[1]Natural Product Informatics Research Center, Korea Institute of Science and Technology, Gangneung 25451, Republic of Korea

[2]Natural Product Research Center, Korea Institute of Science and Technology, Gangneung 25451, Republic of Korea

[3]Department of YM-KIST Bio-Health Convergence, Yonsei University, Wonju, 26493, Republic of Korea

* To whom correspondence should be addressed

Dr. Keunwan Park (keunwan@kist.re.kr)

ZINC5814210 / MEK1

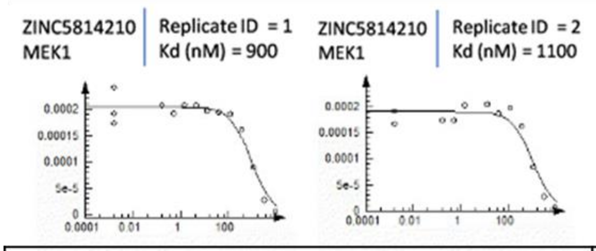

ZINC5814210 / MEK2

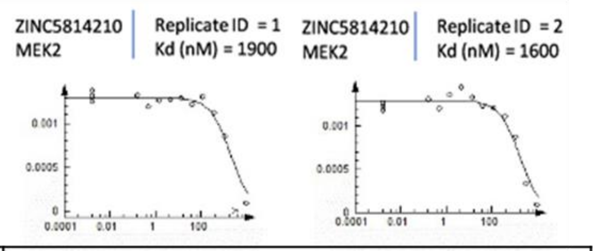

ZINC5814210 / MEK5

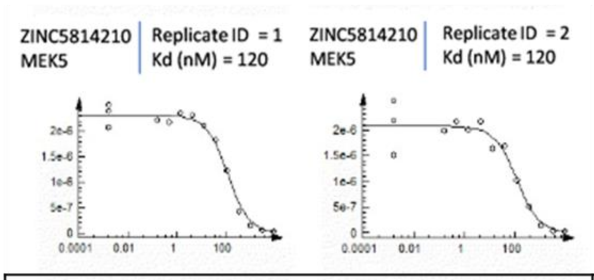

ZINC5479148 / MEK1

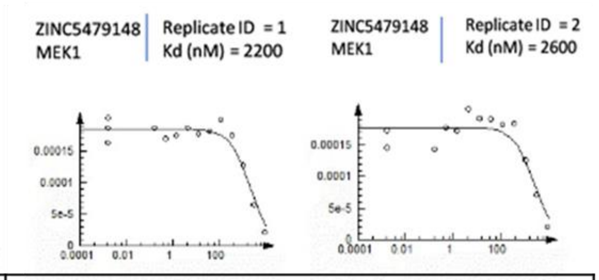

ZINC5479148 / MEK2 *

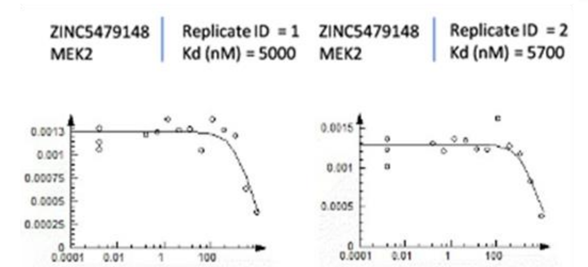

ZINC5479148 / MEK5

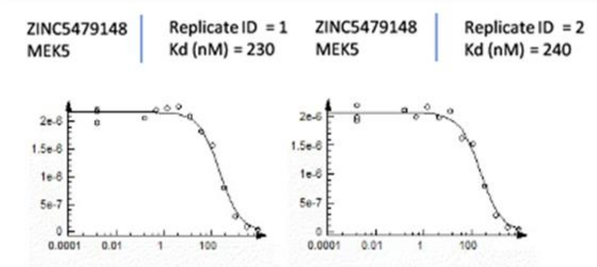

ZINC32911363 / MEK1 *

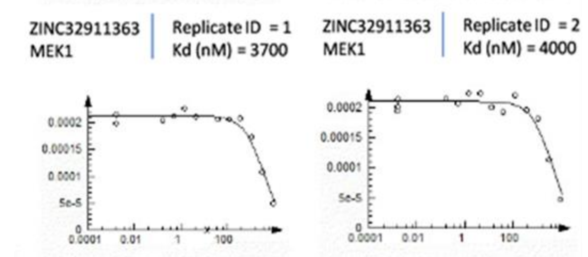

ZINC32911363 / MEK2 *

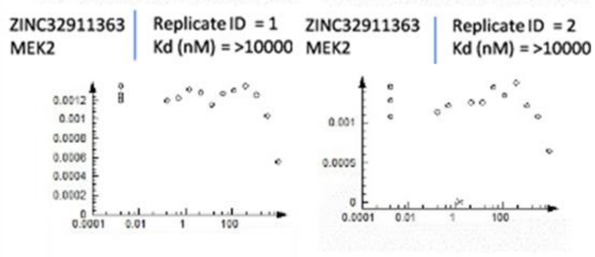

ZINC32911363 / MEK5 *

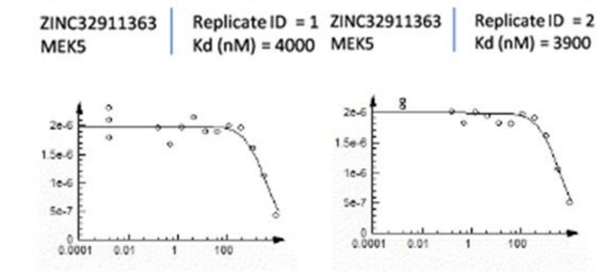

Figure S1. The duplicated dose response curves to determine Kd values of chemical compounds are shown for MEK1, MEK2, and MEK5. X-axis represents ligand concentration (nM) and Y-axis relative inhibitory activity by KdELECT service. We used a maximum 10 µM concentration for all test compounds as a threshold to select active compounds. The dose response curves marked by star(*) represent the case that higher chemical concentration than 10 µM is required to reach a plateau and make more accurate Kd determination.

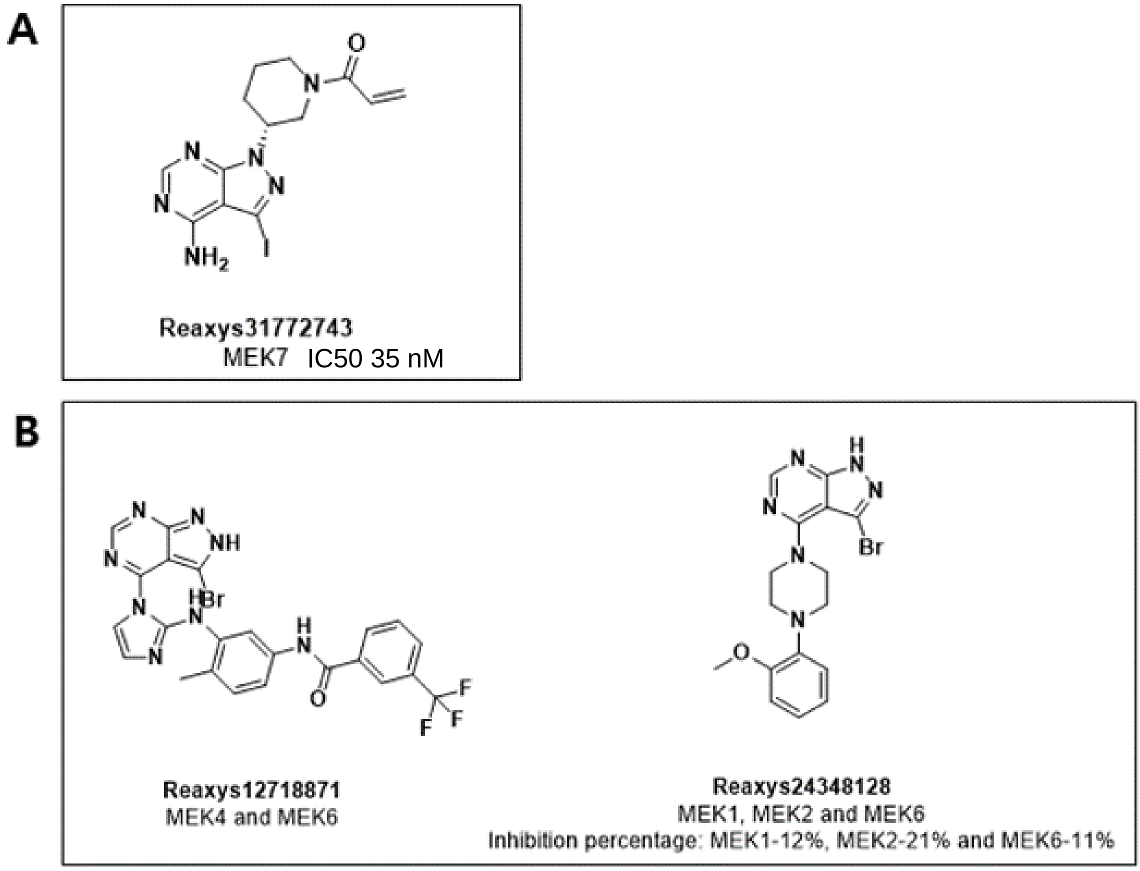

Figure S2. Structurally similar molecules were identified via substructure search in Reaxys database. The molecules that had common or similar substructures as that of ZINC5814210 or ZINC5479148 were selected using the database. (A) Search results when used ZINC5814210 as a query and (B) results when used ZINC5479148 as a query. The MEKs that have activity-related records (inhibition percentage or IC50) with the chemicals are annotated below the chemical IDs. For Reaxys31772743, the IC50 is 35 nM for MEK7. For Reaxys12718871, Reaxys only showed inhibitor records in the ‘substance action on target’ column without specific activity values for MEK4 and MEK6. For Reaxys24348128, the inhibition percentage values for MEK1, MEK2, and MEK6 at 1 µM were reported (SelectScreen^{TM} profiling result, PMID: 19465931). None of the similar compounds showed MEK5-related activities.

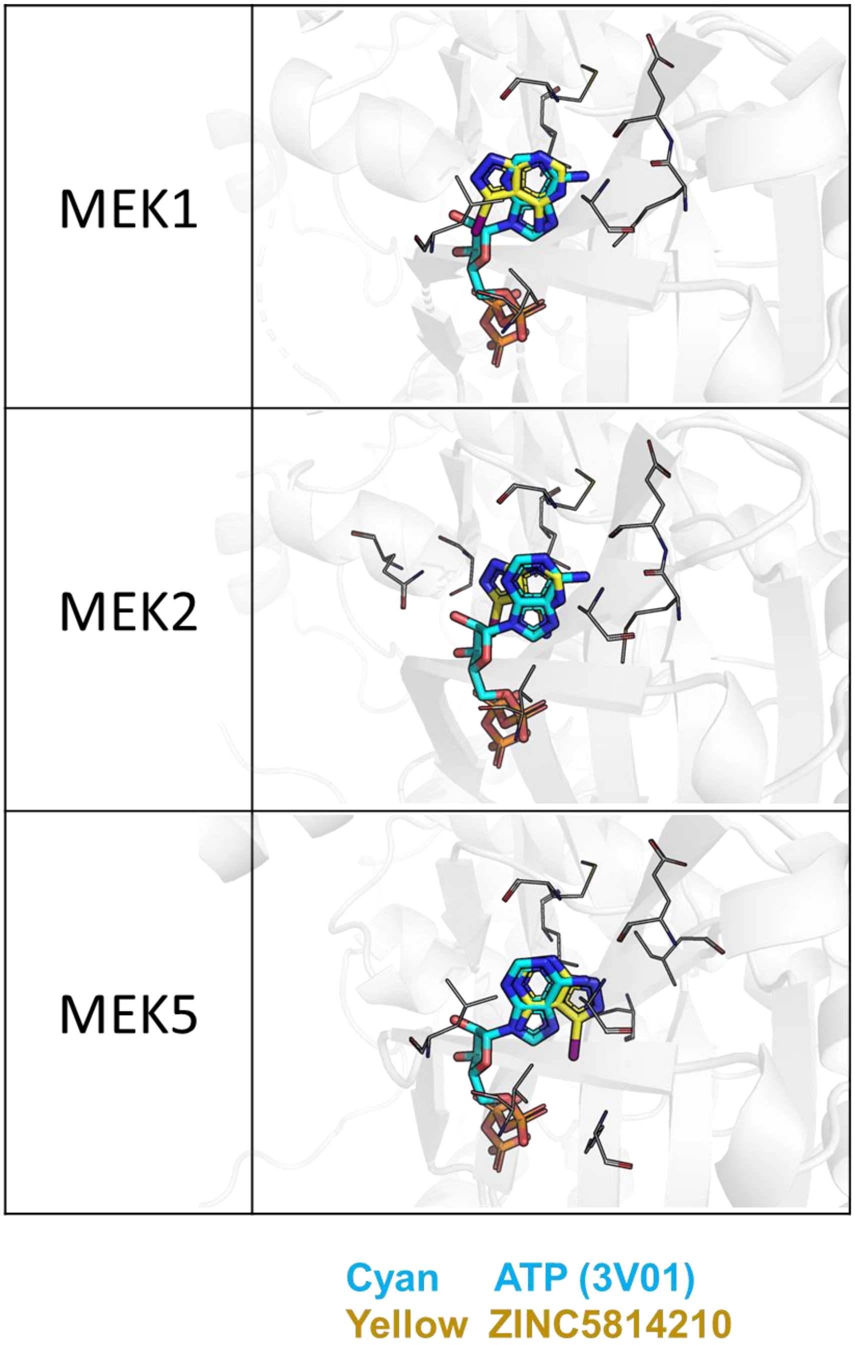

Figure S3. Molecular docking conformations of ZINC5814210 for MEK1, MEK2, and MEK5 are superimposed with ATP found in the MEK1 structure (PDB ID: 3V01). The figure format and perspective are identical to Figure 3 to show the alignment with ATP. For clarity, the binding residues are shown by lines and the ligands by sticks.

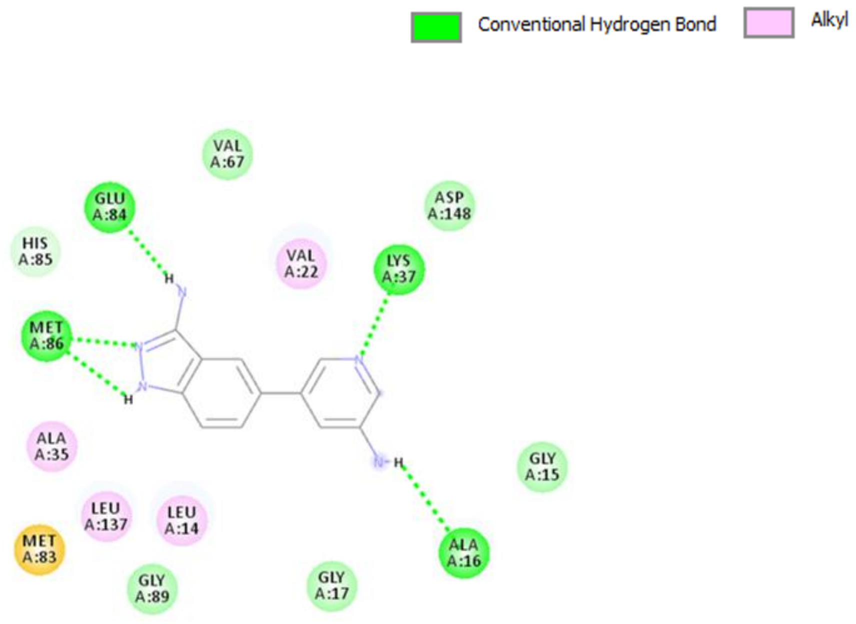

18864755-MEK1

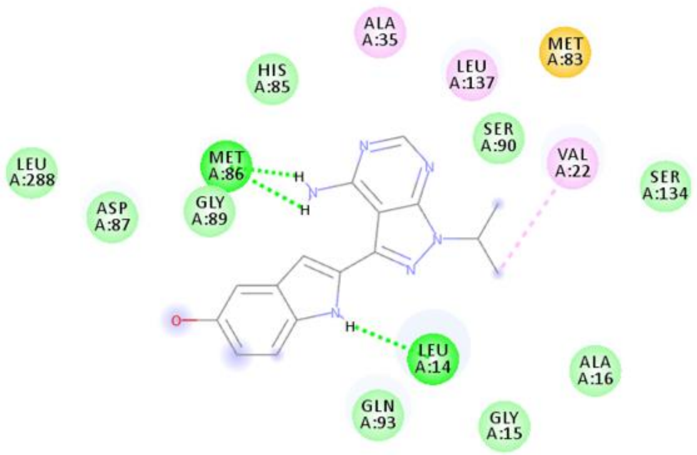

CID24905147-MEK1

Figure S4. Two-dimensional interaction diagram of previously reported MEK1 inhibitors retrieved by 2D fingerprint similarity.

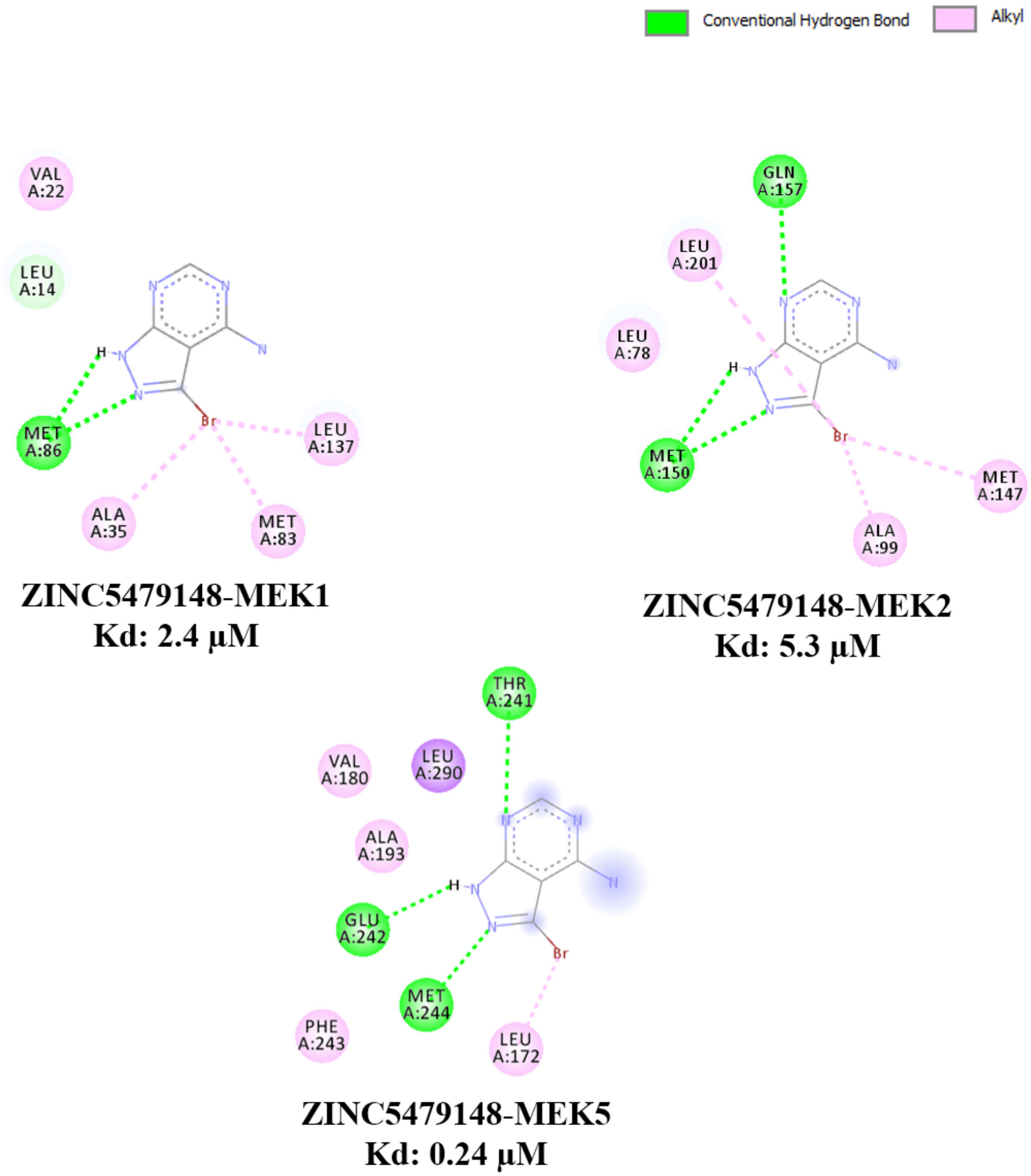

Figure S5. Two-dimensional interaction diagram of MEK-ZINC5479148 docking models.

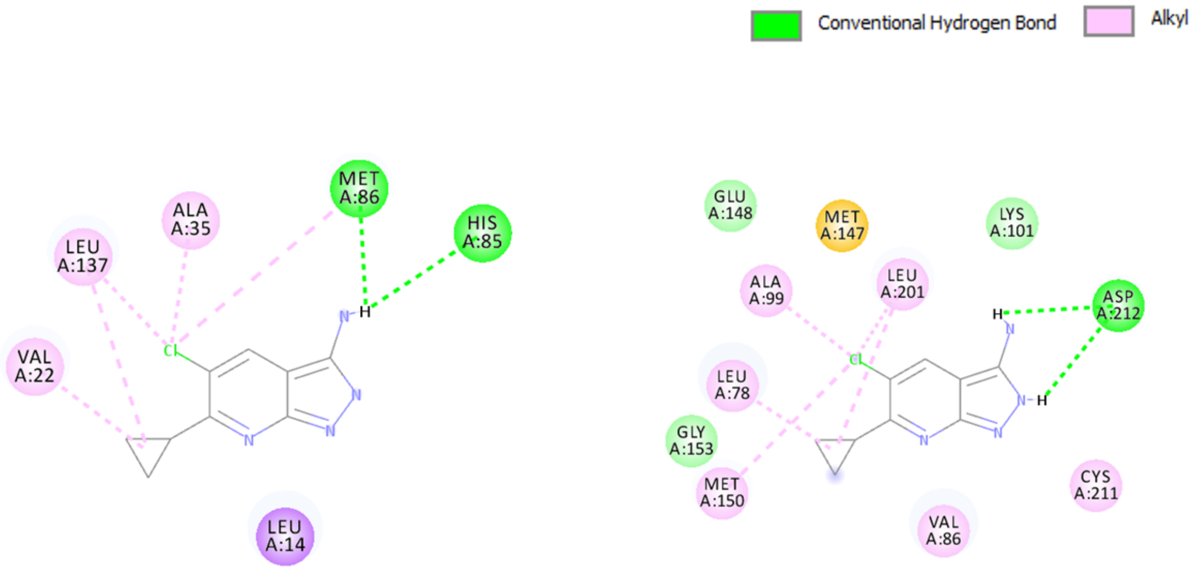

ZINC32911363-MEK1
Kd: 3.8 μM

ZINC32911363-MEK2
Kd: >10 μM

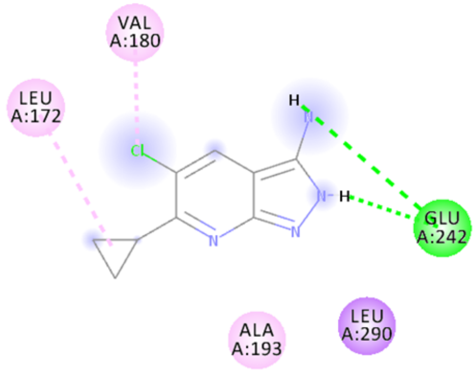

ZINC32911363-MEK5
Kd: 3.9 μM

Figure S6. Two-dimensional interaction diagram of MEK-ZINC32911363 docking models. Except MEK2, ZINC32911363 has better Kd binding affinity to other two MEKs.

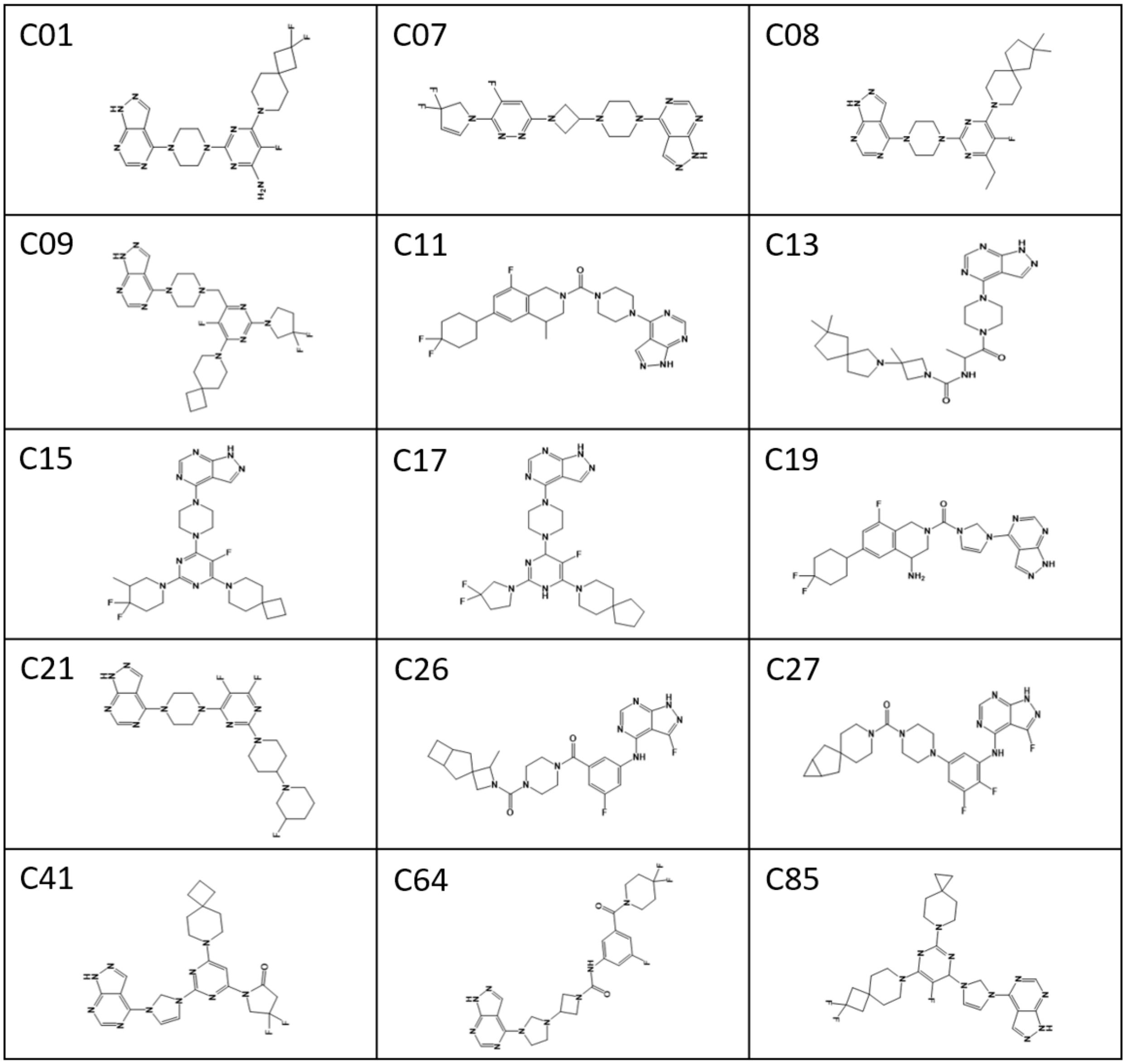

Figure S7. The molecules selected based on binding free energy scores from either MM/GBSA or MM/PBSA or both of the methods.

Table S1. Experimental chemical activity data and cross-validation results (AUC of Precision-Recall curve) for each test target protein. The format of this table is identical to that of Table 1, but the model performance was calculated only for the new active and inactive compounds without considering known active compounds.

		WEE1 (P30291)	MEK1 (Q02750)	EPHB4 (P54760)	TYR (P14679)	Avg. AUC
Chemical Data	Known actives (<100 nM)	19	24	22	22	
	New Exp. Data (Active/Inactive*)	0 / 31	4 / 23	1 / 30	3 / 17	
AUC PR	None	n.a.#	0.275	0.139	0.147	0.187
	$P_{new}P_{prv}$ (PP)	n.a.	0.320	0.179	0.412	0.304
	$N_{new}P_{prv}$ (NP)	n.a.	0.248	0.187	0.127	0.187
	$N_{new}N_{prv}$ (NN)	n.a.	0.316	0.159	0.155	0.210
	PP-NP	n.a.	0.344	0.244	0.482	0.357
	NP-NN	n.a.	0.290	0.191	0.141	0.207
	PP-NN	n.a.	0.336	0.199	0.482	0.339
	PP-NP-NN	n.a.	0.362	0.251	0.491	0.368

* Chemical compounds with lower than POC 20% and higher than 80% are defined as active and inactive compounds, respectively.

AUC values for WEE1 are not available because of the absence of newly identified active compounds from the initial screening results.

Table S2. Comparison of the prediction performance of the standard single chemical-based Random Forest model with the ECBS model trained with PP-NP-NN data.

AUC (PR)	Known Actives (<100nM) New Exp. Data (Active/Inactive)	All		Known Actives		New Exp. Data	
		Single	ECBS	Single	ECBS	Single	ECBS
WEE1 P30291	19 0 / 31	0.925	**0.963**	0.927	**0.970**	NA	NA
MEK1 Q02750	24 4 / 23	0.942	**0.975**	0.966	**0.984**	0.539	**0.711**
EPHB4 P54760	22 1 / 30	0.901	**0.953**	0.899	**0.958**	**1.000**	0.966
TYR P14679	22 3 / 17	**0.979**	0.971	**0.985**	**0.985**	0.468	**0.563**

The chemical screening performance of the ECBS model retrained with PP-NP-NN data (*ECBS*) is compared with that of a typical Random Forest (RF) model based on individual compounds (*Single*). The typical RF model was newly built with identical training data used to train the ECBS model but based on single chemical structures. Thus, it is a binary classification model trained using individual compounds instead of chemical pairs. All training and test data were identical for the two models, although the basal data format was different: chemical pairs vs. individual chemicals. To compare its prediction accuracy with that of the ECBS model, we designed a simple scoring scheme to rank individual compounds by ECBS scores ; the maximum value among the ECBS scores assigned to the known active compounds was considered the final score for each compound in the test set. The test data were divided into three categories: 1) Known Actives (test only for the original data), 2) New Exp. Data: Test only for the new experimental data used to retrain the ECBS model and 3) all tests for both. The AUC PR values were used to estimate the

prediction performance. Chemical compounds with lower than POC 20% and higher than 80% are defined as active and inactive compounds, respectively.

Table S3. Estimation of chemical pair data size

**Data**	P_{prv}	P_{new}	N_{prv}	N_{new}	PP	NP	NN
**Ratio**	p	1	4p	N	p	n(p+1)	4np

The chemical compounds are labeled as P_{new} (new active), P_{prv} (previous active), N_{new} (new inactive), and N_{prv} (previous random inactive data). Among PP, NP, and NN, the data size of the NN is generally the largest because inactive data are usually much more abundant than active data after experimental validation. In addition, to train the ECBS models, the parameter defining the number of random compounds is set to be four times larger than the number of active compounds. The parameter might be reduced by fine-tuning the model performance; however, empirically, including more random data is preferred to represent diverse inactive compounds.

With the following two simplified assumptions: 1) the number of new active compounds (P_{new}) is very small and the majority of the experimental data are inactive, and 2) the random compounds (N_{prv}) are sampled four times more than the previous active compounds (P_{prv}); the data size and ratio were estimated as follows.

Let,

$P_{new} = 1$ (set 1 to represent a small number of new active compounds, Assumption 1)

$P_{prv} = p$ (fold ratio of known active compounds to P_{new})

$N_{new} = n$ (fold ratio of new inactive compounds to P_{new})

$N_{prv} = 4 \cdot P_{prv} = 4p$ (Assumption 2)

then,

$PP = P_{prv} \cdot P_{new} = p + \mu^*$

μ^* : optional self-pairing of Pnew (= Pnew· (Pnew-1)/2) is added in the present study to give more weight to new active compounds)

$NP = N_{new} \cdot P_{new} + N_{new} \cdot P_{prv} = n(1 + p)$, ($N_{rand} \cdot P_{new}$ is not considered)

$NN = N_{new} \cdot N_{prv} = 4np$

Therefore, the NN has about 3-4 times larger than that of the NP. For example, in MEK1, the ratio of PP, NP, and NN was approximately 1:0.82:4.1 (645:528:2667). However, the ratio for each target changes according to the amount of new experimental data and the sampling parameters. In the present study, the parameters were arbitrarily set without optimization.

Table S4. LogP values for the tested compounds. The consensus LogP values for the compounds are calculated using SwissADME as an indicator of cell permeability. All three MEK1 inhibitors and the positive control (PD98059) are within the modest LogP range (-0.5 < LogP < 5).

Name	Consensus LogP
PD98059	2.62
ZINC5814210	0.64
ZINC5479148	0.62
ZINC32911363	1.83

Table S5. GNINA docking scores for MEKs are shown with biochemical binding affinity data in Table 3. GINIA provides CNN (convolution neural network) affinity score with Autodock Vina docking score. The higher CNN affinity score and lower Autodock Vina score represents better binding.

Compound	MEK1	MEK2	MEK5
**Kd (nM)**			
ZINC5814210	1000	1750	120
ZINC5479148	2400	5350*	235
ZINC32911363	3850*	>10000*	3950*
**CNN score**			
ZINC5814210	4.91	5.11	5.49
ZINC5479148	4.83	5.10	5.40
ZINC32911363	5.28	5.76	5.86
**Autodock VINA score (kcal/mol)**			
ZINC5814210	-5.68	-5.68	-5.81
ZINC5479148	-5.72	-5.60	-5.64
ZINC32911363	-7.08	-6.33	-6.45

Table S6. The target prediction results for ZINC5814210 from Swiss target prediction server.

Target	Common name	Uniprot ID	ChEMBL ID	Target Class
Vascular endothelial growth factor receptor 2	KDR	P35968	CHEMBL279	Kinase
Guanine deaminase	GDA	Q9Y2T3	CHEMBL3129	Hydrolase
Cyclin-dependent kinase 2/cyclin A	CDK2<br>CCNA1<br>CCNA2	P24941<br>P78396<br>P20248	CHEMBL2094128	Other cytosolic protein
CDK9/cyclin T1	CDK9<br>CCNT1	P50750<br>O60563	CHEMBL2111389	Other cytosolic protein
Toll-like receptor (TLR7/TLR9)	TLR9	Q9NR96	CHEMBL5804	Toll-like and Il-1 receptors
MAP kinase-interacting serine/threonine-protein kinase MNK1	MKNK1	Q9BUB5	CHEMBL4718	Kinase
Hematopoietic prostaglandin D synthase	HPGDS	O60760	CHEMBL5879	Transferase
Purine nucleoside phosphorylase	PNP	P00491	CHEMBL4338	Enzyme
Adenosine deaminase	ADA	P00813	CHEMBL1910	Hydrolase
Thrombin	F2	P00734	CHEMBL204	Protease
Carbonic anhydrase II	CA2	P00918	CHEMBL205	Lyase
Dipeptidyl peptidase IV	DPP4	P27487	CHEMBL284	Protease
Carbonic anhydrase VA	CA5A	P35218	CHEMBL4789	Lyase
Serine/threonine-protein kinase AKT2	AKT2	P31751	CHEMBL2431	Kinase
Carbonic anhydrase I	CA1	P00915	CHEMBL261	Lyase
Carbonic anhydrase XII	CA12	O43570	CHEMBL3242	Lyase
Carbonic anhydrase IX	CA9	Q16790	CHEMBL3594	Lyase
Xanthine dehydrogenase	XDH	P47989	CHEMBL1929	Oxidoreductase
Arachidonate 15-lipoxygenase	ALOX15	P16050	CHEMBL2903	Enzyme
Epidermal growth factor receptor erbB1	EGFR	P00533	CHEMBL203	Kinase
DNA-dependent protein kinase	PRKDC	P78527	CHEMBL3142	Kinase
PI3-kinase p110-alpha subunit	PIK3CA	P42336	CHEMBL4005	Enzyme
PI3-kinase p110-delta subunit	PIK3CD	O00329	CHEMBL3130	Enzyme
PI3-kinase p110-gamma subunit	PIK3CG	P48736	CHEMBL3267	Enzyme
Tyrosine-protein kinase ABL	ABL1	P00519	CHEMBL1862	Kinase
Protein kinase C alpha	PRKCA	P17252	CHEMBL299	Kinase
Adenosine A2a receptor	ADORA2A	P29274	CHEMBL251	Family A G protein-coupled receptor
Glycogen synthase kinase-3 beta	GSK3B	P49841	CHEMBL262	Kinase

Serine/threonine-protein kinase Aurora-B	AURKB	Q96GD4	CHEMBL2185	Kinase
Serine/threonine-protein kinase Sgk1	SGK1	O00141	CHEMBL2343	Kinase
Casein kinase I delta	CSNK1D	P48730	CHEMBL2828	Kinase
MAP kinase signal-integrating kinase 2	MKNK2	Q9HBH9	CHEMBL4204	Kinase
BR serine/threonine-protein kinase 2	BRSK2	Q8IWQ3	CHEMBL4574	Kinase
Dual-specificity tyrosine-phosphorylation regulated kinase 3	DYRK3	O43781	CHEMBL4575	Kinase
Interferon-induced, double-stranded RNA-activated protein kinase	EIF2AK2	P19525	CHEMBL5785	Kinase
Tyrosine-protein kinase LCK (by homology)	LCK	P06239	CHEMBL258	Kinase
Adenosine A1 receptor	ADORA1	P30542	CHEMBL226	Family A G protein-coupled receptor
Adenosine A3 receptor	ADORA3	P0DMS8	CHEMBL256	Family A G protein-coupled receptor
Serotonin 2a (5-HT2a) receptor	HTR2A	P28223	CHEMBL224	Family A G protein-coupled receptor
Serotonin 2c (5-HT2c) receptor	HTR2C	P28335	CHEMBL225	Family A G protein-coupled receptor
Tyrosine-protein kinase SRC	SRC	P12931	CHEMBL267	Kinase
Kinesin-1 heavy chain/ Tyrosine-protein kinase receptor RET	RET	P07949	CHEMBL2041	Kinase
Tyrosine-protein kinase receptor FLT3	FLT3	P36888	CHEMBL1974	Kinase

Table S7. The target prediction results for ZINC5814210 from Structure Ensemble Approach (SEA) server.

Target ID	P-Value	Max Tc	Cut Sum	Z-Score	Name	Description
ADK_HUMAN	6.42E-13	0.381	4.9826	21.4399	ADK	Adenosine kinase
ADK_RAT	8.08E-50	0.4211	8.2093	87.6866	ADK	Adenosine kinase
BRSK2_HUMAN	9.93E-08	0.375	0.375	12.123	BRSK2	Serine/threonine-protein kinase BRSK2
LIMK1_HUMAN	7.36E-06	0.3243	2.7122	8.7656	LIMK1	LIM domain kinase 1
NR2E3_HUMAN	3.07E-06	0.3953	1.1153	9.4476	NR2E3	Photoreceptor-specific nuclear receptor
PRKDC_HUMAN	2.38E-09	0.4444	6.5712	15.0309	PRKDC	DNA-dependent protein kinase catalytic subunit
